# Molecular mechanisms of disinfectant resistance in *Klebsiella pneumoniae*

**DOI:** 10.1093/jacamr/dlaf247

**Published:** 2026-01-16

**Authors:** Daniel J Noel, Alistair Bailey, Benjamin I Nicholas, Paul Skipp, C William Keevil, Sandra A Wilks

**Affiliations:** School of Biological Sciences, University of Southampton, Highfield Campus, Southampton, UK; School of Biological Sciences, University of Southampton, Highfield Campus, Southampton, UK; Centre for Proteomic Research, University of Southampton, Highfield Campus, Southampton, UK; School of Biological Sciences, University of Southampton, Highfield Campus, Southampton, UK; Centre for Proteomic Research, University of Southampton, Highfield Campus, Southampton, UK; School of Biological Sciences, University of Southampton, Highfield Campus, Southampton, UK; Centre for Proteomic Research, University of Southampton, Highfield Campus, Southampton, UK; School of Biological Sciences, University of Southampton, Highfield Campus, Southampton, UK; School of Biological Sciences, University of Southampton, Highfield Campus, Southampton, UK

## Abstract

**Objectives:**

Chemical disinfectants are critical for infection control in healthcare environments and beyond, as exemplified by their vital role during the COVID-19 pandemic. Despite research repeatedly demonstrating that bacteria can develop adaptations that mitigate the efficacy of chemical disinfectants, the underlying molecular mechanisms remain poorly characterized. This study investigates the mechanisms that underpin resistance demonstrated by disinfectant-adapted *Klebsiella pneumoniae* NCTC 13443 samples.

**Methods:**

Resistant samples have previously undergone long-term *in vitro* adaptation via serial passage in increasing concentrations of common disinfectants benzalkonium chloride (BAC), didecydimethylammonium chloride (DDAC), polyhexamethylene biguanide (PHMB), chlorocresol or bronopol. A multi-omics approach was used to conduct in-depth molecular analyses of the adaptations that contribute to resistance.

**Results:**

*K. pneumoniae* adaptation to BAC, DDAC and PHMB was associated with the modification of lipid A causing the reduction of the net-negative charge of the outer surface, lowering the affinity of cationic disinfectants. This mechanism is also used for polymyxin and colistin resistance, highlighting a potential cross-resistance risk. Chlorocresol-adapted *K. pneumoniae* samples demonstrated increased expression of efflux pumps and expression changes linked to biofilm formation. Bronopol resistance was associated with promoting biofilm formation and increased thioredoxin expression to alleviate oxidative stress. Results indicate the potential role of *N*-ethylmaleimide reductase NemA in bronopol resistance via enzymatic degradation.

**Conclusions:**

These findings provide novel insights into how causative pathogens of healthcare-associated infections can adapt to and mitigate the effectiveness of common chemical disinfectants that are relied on globally every day as a critical infection control measure.

## Introduction

Healthcare-associated infections (HAIs) cause hundreds of millions of infections worldwide every year. *Klebsiella pneumoniae* is a common causative pathogen of HAIs, with *Klebsiella* spp. accounting for ∼10% of cases in US hospitals.^1^

The COVID-19 pandemic highlighted our reliance on chemical disinfectants for infection control; a dependence likely to grow with the ever-increasing prevalence of antimicrobial resistance (AMR). While AMR is typically associated with antibiotic resistance, evidence shows bacteria can also develop tolerance and resistance towards disinfectants.^2^ This has resulted in calls for antiseptic stewardship^3^ and contributed to the banning of triclosan and other antimicrobials in the USA. Benzalkonium chloride (BAC) and five other antiseptics are currently under review, partly due to concerns surrounding antiseptic resistance and cross-tolerance.^4^

For clarity, tolerance is defined as the ability of an organism to survive transient exposure to otherwise lethal concentrations of disinfectant, while resistance is as an inherited ability of an organism to survive and grow at otherwise lethal concentrations irrespective of exposure time.^5^

Clinical *K. pneumoniae* samples have shown varying susceptibilities to chlorhexidine,^6^ iodophor^6^ and BAC,^7^ while *in vitro* experiments have demonstrated *K. pneumoniae* tolerance to chlorhexidine,^8^ BAC^7^ and polyhexamethylene biguanide (PHMB) in combination with betaine.^9^ We recently demonstrated the ability for *K. pneumoniae* samples to adapt to otherwise lethal concentrations of quaternary ammonium compounds (QACs) BAC and didecyldimethylammonium chloride (DDAC), the cationic polymer PHMB, the phenol-derivative chlorocresol and the reactive-oxygen species (ROS)-producing bronopol through evolutionary adaptation.^10^ Their characteristics are presented in Table 1, and the respective pre- and post-adaptation MICs in Table 2. These samples were provisionally characterized as disinfectant tolerant, as genotypic adaptations have not been confirmed. Collateral susceptibility was more common that cross-tolerance among these samples, even among disinfectants with similar mechanisms of action (MOA). This is presumably due to the fitness cost of adaptation.

**Table 1.** Summary of characteristics of the disinfectants used in this study

Compound	Cellular target	Antimicrobial mechanism	Applications
BAC	Membrane	Positively charged quaternary nitrogen groups interact with anionic lipids, promoting their own cellular uptake. This interaction facilitates the insertion of hydrophobic tails into the lipid bilayer, disrupting lipid organization and compromising membrane integrity. As a result, low molecular weight substances leak out, the proton motive force is lost, and oxidative phosphorylation becomes uncoupled.^11^	Surface disinfection sprays and wipes, eye/ear drops, burn treatments.
DDAC	Membrane	Positively charged quaternary nitrogen groups interact with anionic lipids, promoting their own cellular uptake. This interaction facilitates the insertion of hydrophobic tails into the lipid bilayer, disrupting lipid organization and compromising membrane integrity. As a result, low molecular weight substances leak out, the proton motive force is lost, and oxidative phosphorylation becomes uncoupled.^11^	Surface disinfection sprays and wipes, sterilization of surgical equipment.
PHMB	Membrane	The biguanide group sequesters anionic lipids, forming homogenous lipid domains that disturb the structural organization of the membrane. This disruption increases membrane permeability and causes leakage of intracellular content.^11^ Evidence suggests PHMB can translocate across the bacterial membrane, condense DNA and inhibit replication.^12^	Surface disinfection sprays and wipes, wound dressings, contact lens cleaning solution, swimming pool cleaners.
Chlorocresol	Membrane	Compromises the permeability barrier through membrane disruption, inducing leakage of low molecular weight components. Causes downstream collapse of proton motive force and uncoupling of oxidative phosphorylation.^13^	Antiseptic, preservative.
Bronopol	Proteins. ROS generated target macromolecular structures	Catalyses oxidation of thiols to disulphides, cross-linking proteins and impeding functionality. This reaction also generates ROS that cause downstream damage to intracellular components.^14^	Disinfectant, preservative.

**Table 2.** Disinfectant MIC values against *K. pneumoniae* NCTC 13443 before and after adaptation

Disinfectant	*K. pneumoniae* NCTC 13443 MIC (mg/L)	
	Pre-adaptation	Post-adaptation
BAC	20	56
DDAC	6	14
PHMB	6	9
Chlorocresol	200	260
Bronopol	8	41

BAC tolerance in *K. pneumoniae* has been attributed to increased efflux pump activity.^7^ However, chemical inhibition of efflux pump activity had no impact on BAC and PHMB susceptibility of *K. pneumoniae* isolates,^15^ suggesting other contributing mechanisms. In other species, porin down-regulation and membrane charge alteration via lipid A modification has been associated with BAC tolerance.^16^ Tolerance to PHMB and bronopol has not been investigated in detail, so any mechanisms of tolerance remain unknown. Chlorocresol tolerance has not been reported or investigated, although studies have shown a link between phenolic disinfectant susceptibility and efflux pump activity.^17^

This study aims to characterize the molecular mechanisms enabling these disinfectant-adapted *K. pneumoniae* samples to survive otherwise lethal disinfectant concentrations via whole-genome sequencing and label-free quantitative proteomics. Addressing these aims will provide valuable insights into how bacteria can adapt to commonly used disinfectants in healthcare, commercial and household environments, while also guiding future decisions on healthcare cleaning routines and antiseptic stewardship policies.

## Materials and methods

### Bacterial strains and growth media

Whole-genome sequencing and proteomic analysis was performed on three biological replicates of disinfectant-adapted *K. pneumoniae* samples generated previously.^10^ Owing to minor colour variations between colonies of BAC-adapted replicates plated on CHROMagar^™^ Orientation chromogenic agar, five samples were analysed to check for technical consistency. For a comprehensive description of the adaptation methodology and initial characterization of the samples, see Noel *et al*.^10^ In brief, samples were passaged daily in Mueller–Hinton broth containing increasing concentrations of disinfectant until no further tolerance developed over 15 consecutive passages.^10^ Pre- and post-adaptation MICs are displayed in Table 2. Total adaptation times varied from 69 to 103 passages.^10^

Adapted samples were cultured overnight at 37°C in Mueller–Hinton broth containing a sub-MIC (post-adaptation) of respective disinfectant (55 mg/L BAC, 13 mg/L DDAC, 8 mg/L PHMB, 240 mg/L chlorocresol, 40 mg/L bronopol)^10^ before protein and DNA extraction. For comparison, three pre-adaptation samples were prepared in the absence of disinfectant.

### Stock solutions of antimicrobial compounds

Here, 10 000 mg/L BAC, DDAC, PHMB and bronopol (Thor Specialities) stocks were prepared in ddH_2_O immediately before use. Chlorocresol (Lanxess) was prepared similarly in undiluted DMSO. Working concentrations of DMSO had no detectable impact on *K. pneumoniae* colony forming unit counts.

### Whole-genome sequencing

One millilitre aliquots were washed in PBS three times before DNA extraction via DNeasy PowerSoil Pro Kit (Qiagen) following the manufacturer’s instructions. Lysates were frozen at −20°C until required. Samples were sequenced by Novogene via Illumina^®^ NovaSeq^™^ 6000.

Raw reads were cleaned to remove adapter contamination, reads with >10% uncertain bases or >50% low quality nucleotides, before mapping to the reference genome with BWA. Variants were called using GATK, compared using bcftools and annotated using the ANNOVAR software tool.

### Global quantitative proteomics

Samples were washed three times in PBS, pelleted and resuspended in lysis buffer [50 mM tris, 150 mM NaCl, 0.1% w/v SDS, cOmplete^™^ protease inhibitor cocktail (Roche)]. After sonication (120 s total, 12% amplitude, 10-s pulses) and centrifugation (12 000g, 20 min, 4°C), protein concentrations were quantified via BCA assay. Lysates were frozen at −20°C until required.

Volumes containing 100 µg of protein were mixed with 600 µL of methanol, 150 µL of chloroform, 450 µL of dH_2_O and subsequently centrifuged (14 000g, 10 min). After the upper aqueous layer was removed, 450 µL of methanol was added before mixing and centrifugation. Protein pellets were air dried before resuspension in 100 µL of 6 M urea, 50 mM tris-HCl, 5 mM dithiothreitol (pH 8.0), incubated for 30 min at 37°C, before incubation with 15 mM iodoacetamide for 30 min at room temperature. Four micrograms trypsin/Lys-C mix (Promega) were added for 4 hours at 37°C, before dilution in 750 µL of 50 mM tris-HCl (pH 8.0) and overnight incubation. Digestion was terminated by the addition of trifluoroacetic acid before centrifugation (14 000g, 10 min). Peptides were purified using Oasis PRiME HLB 96-well µElution plates (Waters) by elution in 70% acetonitrile and spin-dried under vacuum.

Samples were resuspended in 50 µL of 0.1% v/v formic acid before mass spectrometry (UltiMate 3000 RSLC nano system with Orbitrap Fusion^™^ Tribrid^™^ Mass Spectrometer, Thermo Fisher Scientific).

Peptide/protein identification and area under the curve quantification were performed using PEAKS Studio Xpro (Bioinformatics Solutions). Proteome coverage was 25.6%–28.3%. Proteins were filtered to include proteins identified across all parent and adapted replicates. A 1% false discovery rate and minimum ±2 log_2_ fold change threshold were used to define significant, differentially expressed proteins.

### Data analysis

Phylogenetic trees were generated using CSI Phylogeny v.1.4^18^ using default parameters and *K. pneumoniae* NCTC 13443 as the reference genome. FigTree v.1.4.4^19^ was used for visualization.

Genetic variants were filtered to exclude synonymous single nucleotide polymorphisms (SNPs), non-coding mutations and mutations not conserved across all replicates. Gene Ontology (GO) biological function networks were generated using ClueGO v.2.5.9^20^ in Cytoscape v.3.9.1^21^ with *K. pneumoniae* strain 342 as the reference genome. Small variant sets are instead presented in Table 3.

**Table 3.** Conserved mutations detected in all biological replicates of *K. pneumoniae* NCTC 13443 disinfectant-adapted samples (*n* = 3)

Resistant sample	Gene	Variation type	Gene annotation	Protein annotation	Protein product
PHMB	*basS*	ns-SNP	469A>C	T157P	Sensor protein BasS/PmrB
	*kdgR*	ns-SNP	374A>G	D125G	Transcriptional regulator KdgR
Chlorocresol	*marR_1*	fs-ins	78dupT	T27N*fs*X3	DNA-binding transcriptional repressor MarR
	*yicC*	fs-ins	290_291insC	M97I*fs*X4	Protein YicC
	*acrB_5*	ns-SNP	163A>C	I55L	RND efflux system
	*cpdA_2*	ns-SNP	136A>G	S46G	3′,5′-cyclic-nucleotide phosphodiesterase
	*fnr*	ns-SNP	289G>A	D97N	Fumarate and nitrate reduction regulatory protein
	*htrE*	ns-SNP	2233A>C	S745R	Fimbriae usher protein StcC
	NCTC13443_06216	ns-SNP	104T>G	V35G	Fimbrial-like protein
	NCTC13443_06725	ns-SNP	1690G>T	A564S	Membrane protein
	*yjcC_2*	ns-SNP	742C>T	P248S	Cyclic-guanylate-specific phosphodiesterase
	*fim_1*	nfs-del	110_121del	37_41del	Fimbrial protein MrkD
Bronopol	*cpdA_2*	ns-SNP	136A>G	S46G	3′,5′-cyclic-nucleotide phosphodiesterase
	*htrE*	ns-SNP	2233A>C	S745R	Fimbriae usher protein StcC
	NCTC13443_06216	ns-SNP	104T>G	V35G	Fimbrial-like protein
	*purR_2*	ns-SNP	413GT	R138L	Purine nucleotide synthesis repressor
	*putA_3*	ns-SNP	664A>C	T222P	PutAP proline dehydrogenase transcriptional repressor
	*rhaS_2*	ns-SNP	314G>C	R105P	Negative transcriptional regulator of cel operon
	*yjcC_2*	ns-SNP	742C>T	P248S	Cyclic-guanylate-specific phosphodiesterase
	*fim_1*	nfs-del	110_121del	37_41del	Fimbrial protein MrkD

ns-SNP, non-synonymous single nucleotide polymorphism; fs-ins, frameshift insertion; nfs-del, non-frameshift deletion; RND, resistance nodulation division; NADP, nicotinamide adenine dinucleotide phosphate; GTP, guanosine triphosphate.

GO enrichment of differentially expressed proteins was performed using the Database for Annotation, Visualization and Integrated Discovery (DAVID)^22,23^ with *K. pneumoniae* MGH 78578 as the background list. Enriched biological process and cellular component GO terms with a *P* value of ≤0.05 were considered significant and visualized as heatmaps via GraphPad Prism v.9.4.1. Network maps of differentially expressed proteins were generated using ClueGO^20^/Cytoscape^21^ and arranged by Kyoto Encyclopedia of Genes and Genomes (KEGG) biological pathways annotations, with *K. pneumoniae* strain 342 used as reference.

## Results and discussion

### Characterization of disinfectant-adapted *K. pneumoniae* samples

**Figure 1.**
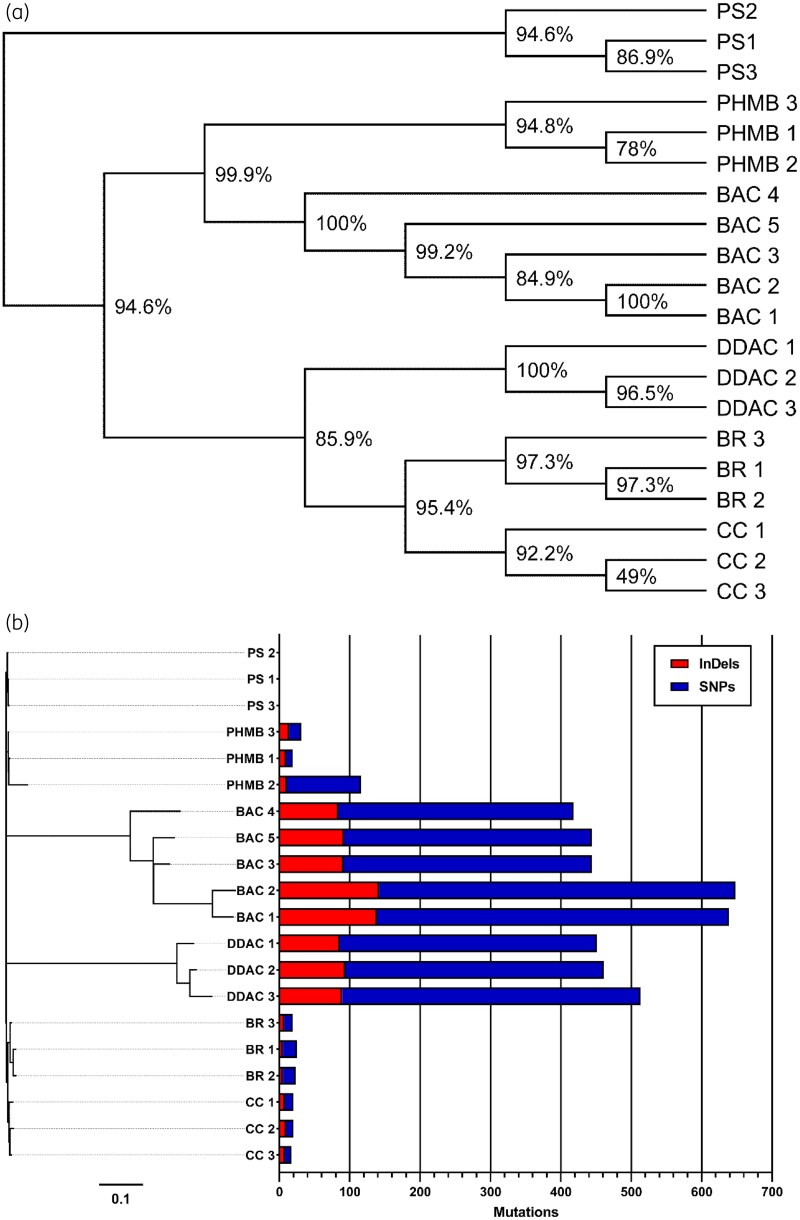
Genetic characterization of *Klebsiella pneumoniae* NCTC 13443 disinfectant-adapted samples. (a) Unrooted, unscaled phylogenetic tree resistant samples. Node values indicate bootstrap values as a percentage. (b) The total number of mutations acquired by *Klebsiella pneumoniae* NCTC 13443 disinfectant-adapted samples compared to the respective untreated parent samples. The phylogram shows the relative genetic relationships of the samples, with the distance scale indicating the number of nucleotide substitutions per site. InDels, insertions or deletions that are ≤50 base pairs in length. PS: parent samples. BR, bronopol-adapted samples; CC, chlorocresol-adapted samples.

Unrooted phylogenetic trees (Figure 1) group samples into clades by disinfectant treatment, showing that adapted samples have acquired conserved genotypic adaptations facilitating survival in the presence of otherwise lethal concentrations of disinfectant (Table 2) irrespective of exposure time.^10^ We therefore classify the samples as resistant, although only to concentrations of disinfectants significantly lower (Table 2) than those at point of use in commercial products, which are typically in the 10^2^–10^3^ mg/L range.^24,25^ Despite this, it is still pertinent to establish what mechanisms are being used to mitigate the efficacy of disinfectants, especially considering how various factors including dilution factor,^26^ organic load,^27^ exposure time^28^ and residual compound degradation^29^ can effectively reduce the exposure concentration.

Disinfectant resistant samples displayed short genetic distances from each other, except for QAC-adapted (BAC, DDAC) samples (Figure 1b). Genetic distances between adapted clusters did not align with MOA similarities, with BAC- and DDAC-adapted clades being furthest apart despite both being cationic membrane-active agents with a near-identical general MOA. This suggests small differences in interactions with components of the membrane can manifest distinct adaptations. Although sharing few mutations in common, BAC-adapted samples previously demonstrated cross-resistance to DDAC^10^ implying overlapping mechanisms.

QAC-adapted samples demonstrated a uniquely high number of mutations (Figure 1b), indicating a strong selection pressure. Conversely, PHMB, chlorocresol and bronopol-adapted samples showed fewer mutations and shorter genetic distances between them, despite more varied MOAs. This shows that few mutations are required for *K. pneumoniae* to adapt to these agents, and suggests that the strength of the selection pressure had more influence on the genotype than MOA similarities.

PHMB-adapted sample 2 accumulated many unique mutations compared with other replicates, indicating inconsistent resistance mechanisms (Figure 1b). PHMB-adapted samples also showed varying cross-resistance profiles,^10^ thought to be due to multiple suggested target sites. While PHMB is primarily regarded as membrane-active, research suggests alternative action via condensing of nucleic acids.^12^ Multiple target sites would allow for different resistance strategies, explaining the lack of homogeneity between the biological replicates. Despite this, all PHMB-adapted replicates remained grouped in a single clade, indicating conserved adaptations.

**Figure 2.**
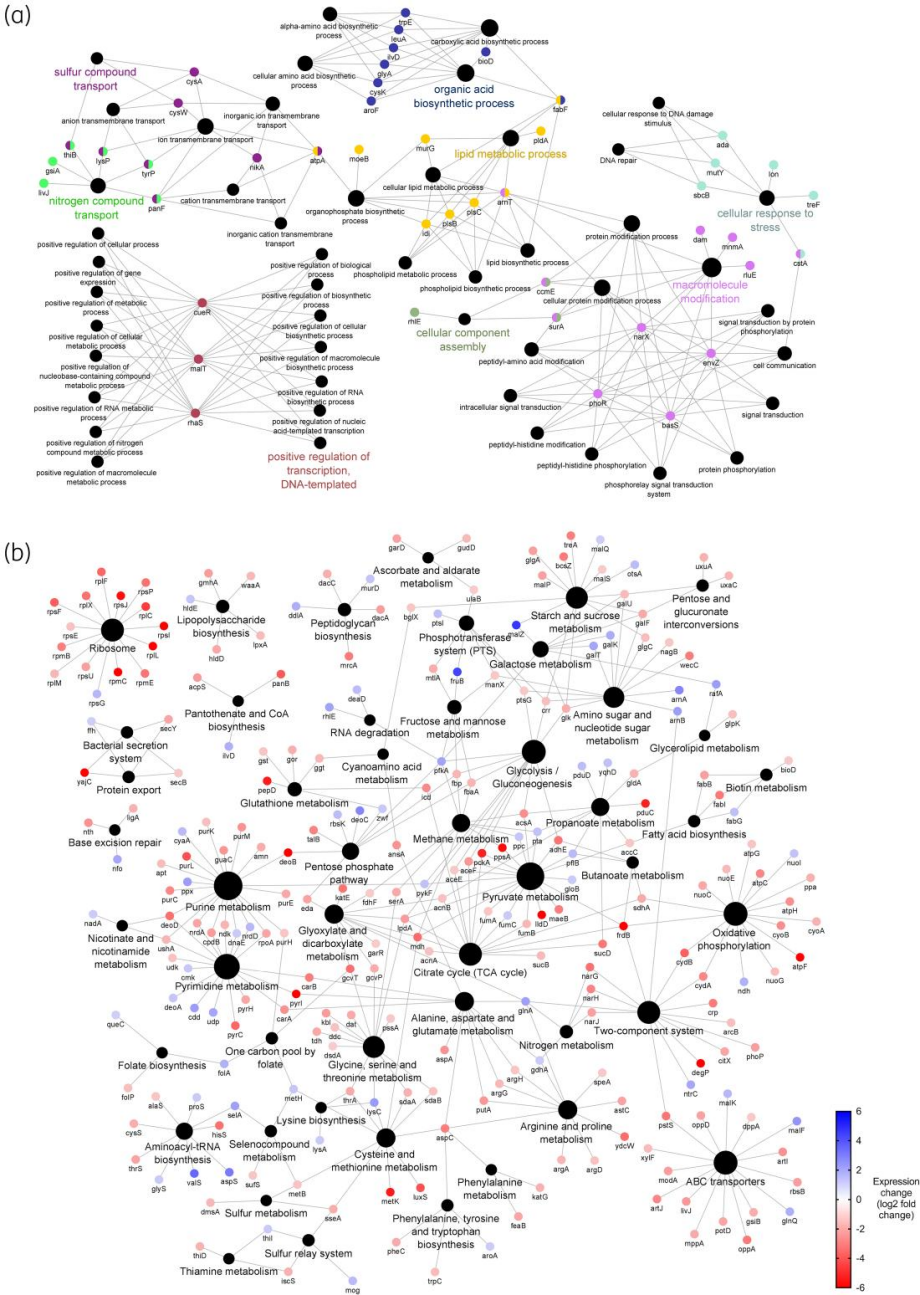
Genomic and proteomic characterization of *Klebsiella pneumoniae* NCTC 13443 benzalkonium chloride-adapted samples. (a) Network diagram of genes that contain conserved mutations across all replicates (*n* = 5). Genes are indicated by the coloured dots, arranged according to GO biological process annotation. Colours indicate biological process annotation. Black dots indicate biological process annotations, as labelled. Lines connect genes to their annotations. (b) Network diagram of differentially expressed proteins (coloured dots), arranged according to KEGG pathway annotation. Node labels show associated gene name. Blue and red coloration indicates increased or reduced expression, respectively. Black dots indicate KEGG pathway annotations, with size proportional to number of associated differentially expressed proteins. Lines connect genes to their annotations.

**Figure 3.**
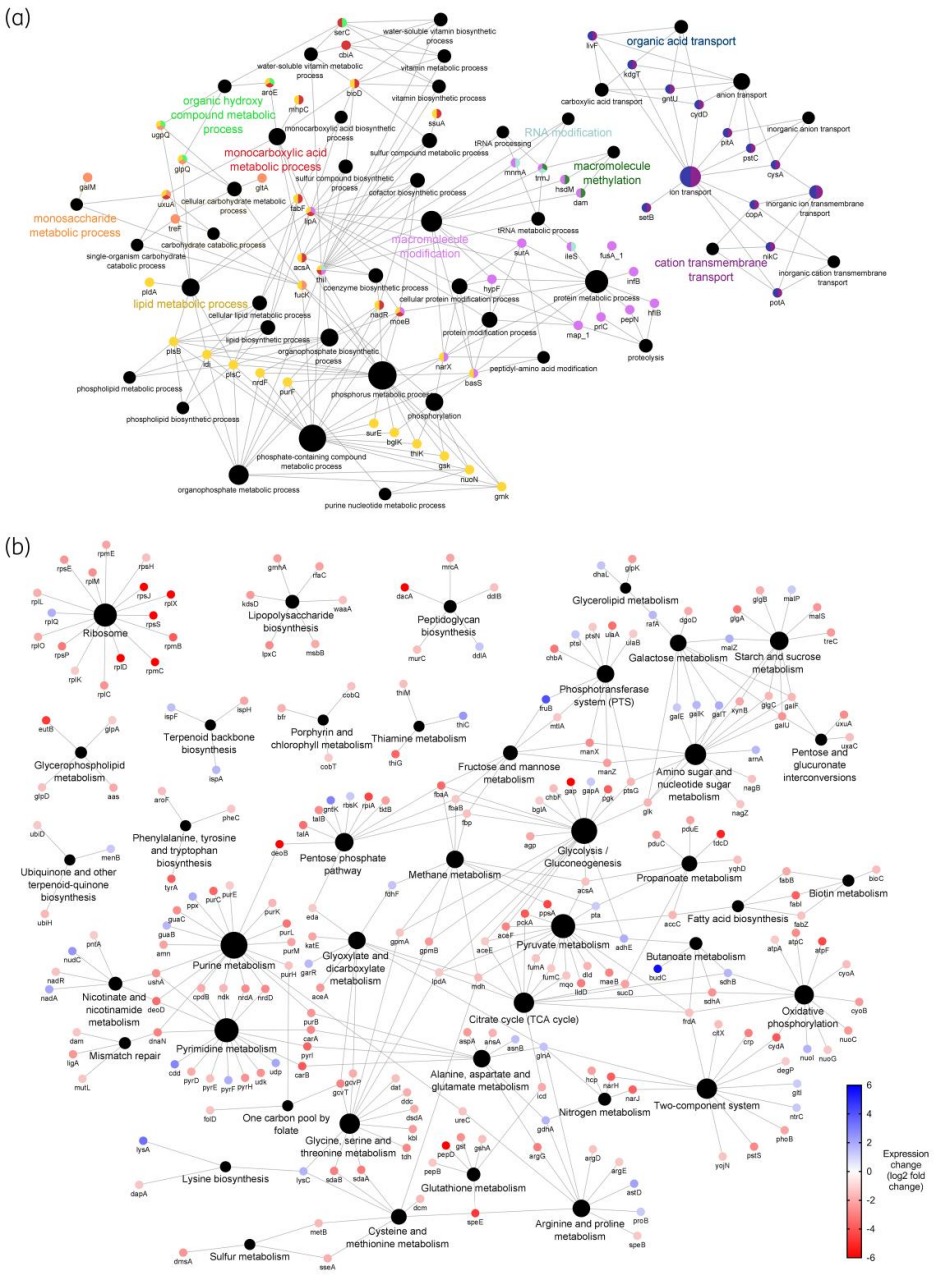
Genomic and proteomic characterization of *Klebsiella pneumoniae* NCTC 13443 didecyldimethylammonium chloride-adapted samples. (a) Network diagram of genes that contain conserved mutations across all replicates (*n* = 3). Genes are indicated by the coloured dots, arranged according to GO biological process annotation. Colours indicate biological process annotation. Black dots indicate biological process annotations, as labelled. Lines connect genes to their annotations. (b) Network diagram of differentially expressed proteins (coloured dots), arranged according to KEGG pathway annotation. Node labels show associated gene name. Blue and red coloration indicates increased or reduced expression, respectively. Black dots indicate KEGG pathway annotations, with size proportional to number of associated differentially expressed proteins. Lines connect genes to their annotations.

**Figure 4.**
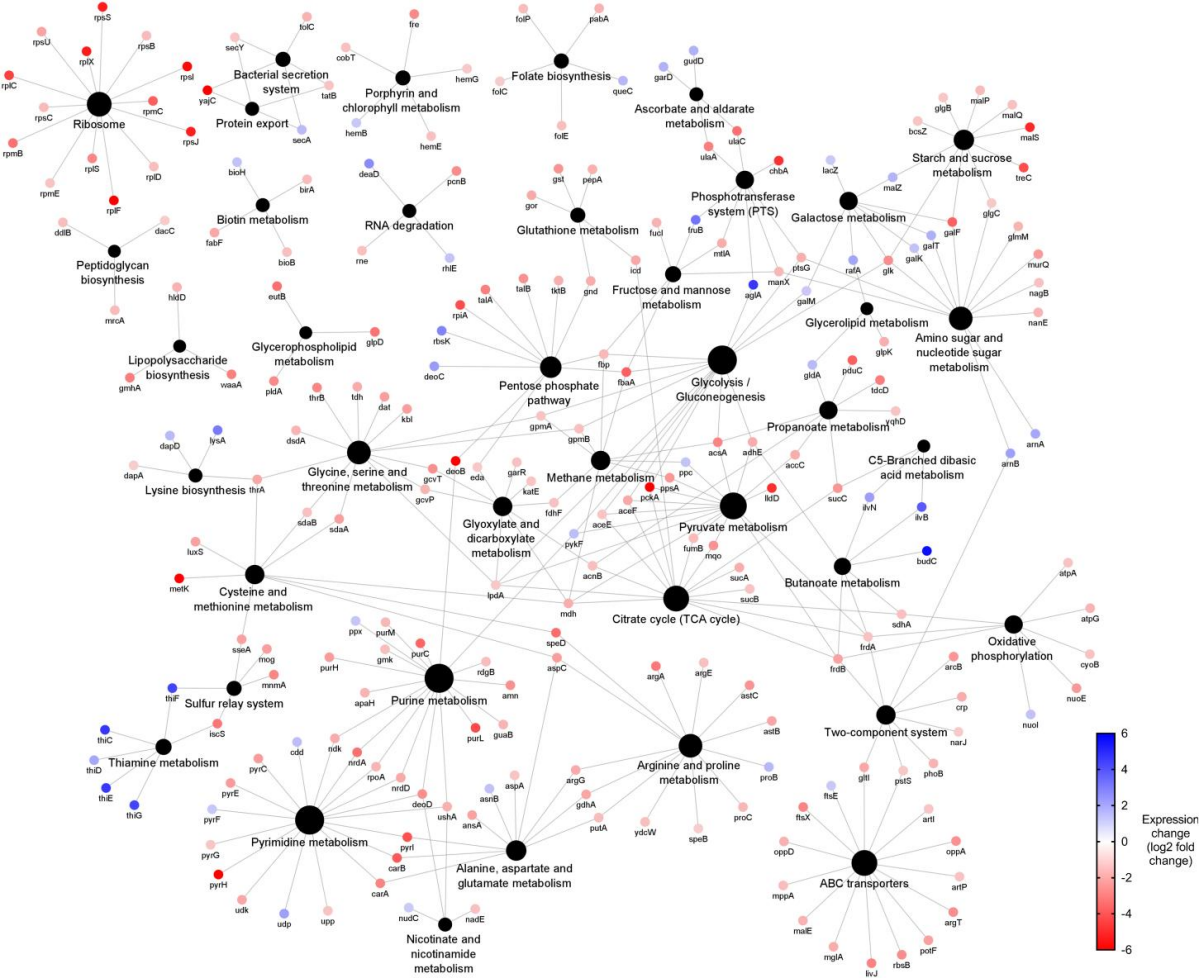
Network diagram of differentially expressed proteins in polyhexamethylene biguanide-adapted *Klebsiella pneumoniae* NCTC 13443 samples. Differentially expressed proteins are indicated by the coloured dots, arranged according to KEGG pathway annotation. Blue and red coloration indicates up or down expression, respectively. Black dots indicate KEGG pathway annotations, as labelled. Lines connect proteins to their annotations. This network map was generated by Cytoscape v.3.9.1 using the ClueGO v.2.5.9 plugin.

**Figure 5.**
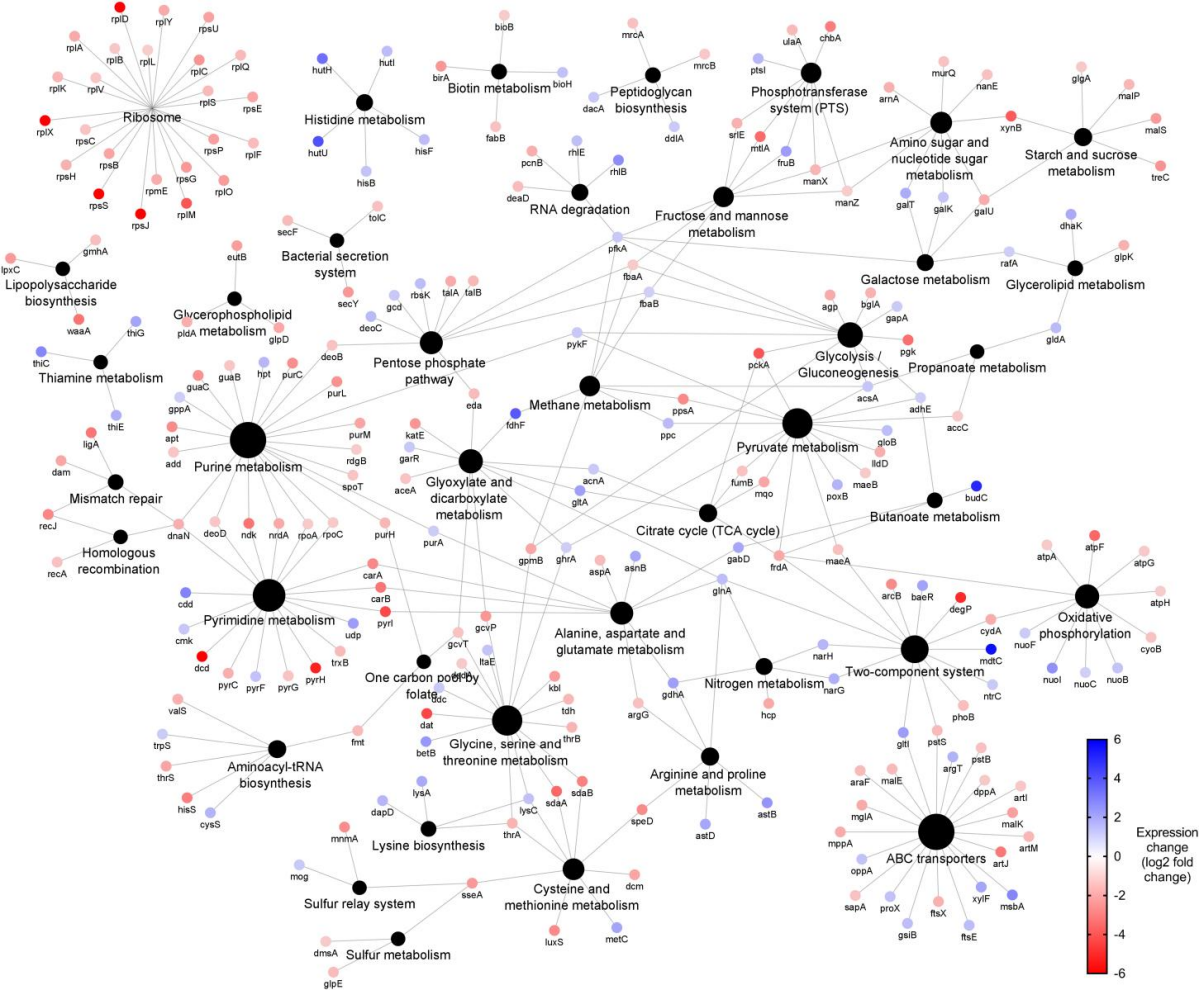
Network diagram of differentially expressed proteins in chlorocresol-adapted *Klebsiella pneumoniae* NCTC 13443 samples. Differentially expressed proteins are indicated by the coloured dots, arranged according to KEGG pathway annotation. Blue and red coloration indicates up or down expression, respectively. Black dots indicate KEGG pathway annotations, as labelled. Lines connect proteins to their annotations. This network map was generated by Cytoscape v.3.9.1 using the ClueGO v.2.5.9 plugin.

**Figure 6.**
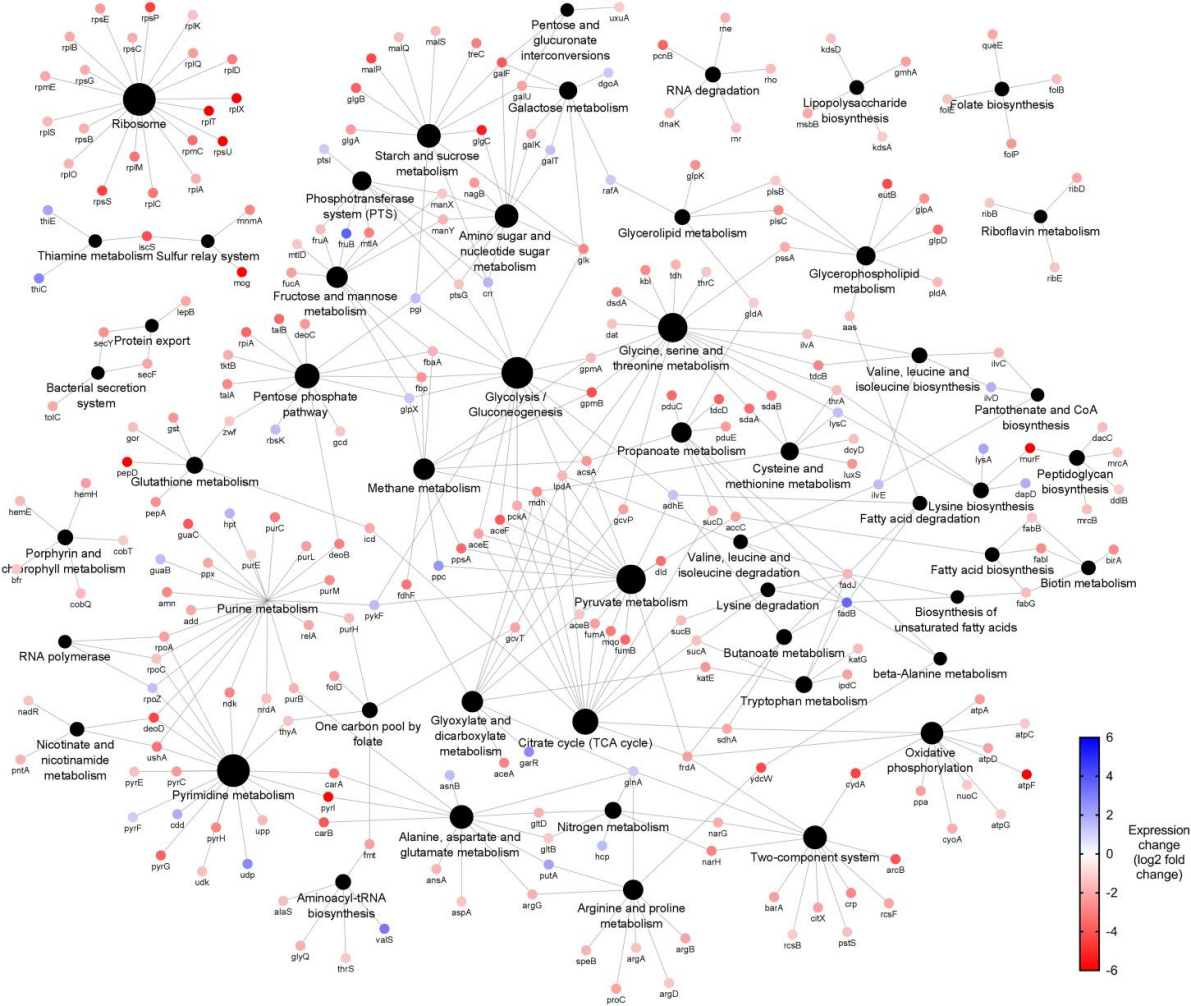
Network diagram of differentially expressed proteins in bronopol-adapted *Klebsiella pneumoniae* NCTC 13443 samples. Differentially expressed proteins are indicated by the coloured dots, arranged according to KEGG pathway annotation. Blue and red coloration indicates up or down expression, respectively. Black dots indicate KEGG pathway annotations, as labelled. Lines connect proteins to their annotations. This network map was generated by Cytoscape v.3.9.1 using the ClueGO v.2.5.9 plugin.

Quantitative proteomics showed lower expression across all adapted samples (Figures 2b, 3b, 4, 5, 6), with reduced enrichment of arginine biosynthesis, gluconeogenesis, translation and TCA cycle biological process GO terms (Figure S1, available as Supplementary data at *JAC-AMR* Online), indicating reduced growth and metabolism. This is characteristic of dormant phenotypes, which classically show reduced antimicrobial susceptibility.^30,31^ This also demonstrates the fitness cost of adaptation, with energy being diverted to maintain a resistant phenotype.

### *K. pneumoniae* resistance to QACs

Mutated genes in QAC-adapted samples were linked to lipid metabolic and macromolecule modification cellular processes (Figures 2a and 3a). Both BAC and DDAC-adapted samples contained a conserved non-synonymous SNP in histidine kinase *basS* (polymyxin resistance protein B, PmrB), which positively regulates *arnABCDEFT* genes responsible for 4-amino-4-deoxy-L-arabinose (L-Ara4N) synthesis. ArnT modifies lipid A with L-Ara4N, neutralizing the negative charge of the 4′-phosphate group and decreasing the net-negative charge of the outer leaflet.^32^ This reduces the affinity of cationic peptides including colistin and polymyxin B, facilitating resistance.^32^

The mutation causes a A68V substitution within the transmembrane region (Tables S1 and S2), responsible for physiological signal detection and conformational changes activate *arnABCDEFT* genes.^33^ Similar mutations in this region constitutively activate *E. coli* PmrB and cause increased expression of *arnT*.^34^ Proteomics reveals increased expression of downstream ArnAB in BAC-adapted samples (Figure 2), and ArnA in DDAC-adapted samples (Figure 3), suggesting the A68V substitution constitutively activates BasS.

BAC-adapted samples contained a conserved N457S substitution in *arnT* (Table S1) in transmembrane domain helix 13 adjacent to the lipid A binding cavity, specifically the polar region that associates with 3-deoxy-D-manno-oct-2-ulosonic acid sugars.^35^ Asparagine and serine are polar uncharged residues, so this relatively subtle substitution may alter lipid A binding affinity and contribute to BAC resistance.

BAC-adapted samples also displayed a conserved V187M substitution in the linker region of histidine kinase *envZ*. This protein regulates OmpF/C expression via the activation of OmpR in response to changes in osmolarity. No downstream proteins were detected via proteomics due to protein coverage limitations, so the impact remains unclear. The absence of this mutation in DDAC-adapted samples suggests it is unique to BAC.

Both BAC and DDAC-adapted samples contained mutations in DNA repair genes *ada* and *mutY*, while BAC also had a mutation in *sbcB*. DDAC-adapted samples showed decreased expression of methyl-directed mismatch repair system protein MutL. Loss of DNA repair function contributes to hypermutable phenotypes,^36^ allowing for greater stochastic development of beneficial mutations and explaining the large number of QAC-associated mutations.

Regarding efflux pumps, all QAC-adapted samples carried an E130K substitution in AcrB, the inner membrane component of multidrug efflux pump AcrAB-TolC. Located in the PN1 subdomain of the porter domain where both proximal and distal binding pockets are located,^37^ similar substitutions can alter substrate specificity,^38^ resulting in AMR.^39^ DDAC-adapted samples also had conserved substitutions in resistance-nodulation-division family efflux pumps MdtC and BepE, plus a F81S substitution proximal to the ligand binding site of AcrAB regulator AcrR.^40^ BAC-adapted samples showed increased AcrAB and BepE expression, further reinforcing the importance of efflux pumps in QAC resistance.

DDAC resistance appeared to require additional efflux pump modifications, probably reflecting necessary modifications to substrate specificity. This explains the lack of reciprocation in cross-resistance profiles between BAC and DDAC-adapted samples.^10^

Collectively, *K. pneumoniae* QAC resistance occurs via modification of lipid A with L-Ara4N, lowering cell surface negative charge and reducing affinity of cationic QACs. As this is a well-established mechanism facilitating resistance to cationic peptides including polymyxin B and colistin, it is highly likely that this mechanism confers cross-resistance. QAC resistance is also associated with efflux pump activity, with DDAC requiring additional efflux modifications compared with BAC. Adaptation is further supported by reduced DNA repair functionality, giving rise to a hypermutable *K. pneumoniae* phenotype.

### *K. pneumoniae* resistance to PHMB

Similar to QAC-adaptation, molecular analysis of PHMB-adapted *K. pneumoniae* showed links to lipid A modification. PHMB-adapted *K. pneumoniae* samples displayed only two conserved mutations across all biological replicates, in *basS* and *kdgR* (Figure 4, Table 3, Table S3). The BasS adaptation causes T157P in the dimerization and histidine phosphotransferase domain, responsible for phosphotransferase, phosphatase and autokinase activities. As this domain modulates protein activity, and expression of downstream ArnA and ArnB was increased (Figure 4), this mutation probably increases activity or constitutively activates BasS.

Lipid A modification was also seen in BAC- and DDAC-adapted samples and is known to underpin polymyxin and colistin resistance.^41^ As this exact mutation has previously been attributed with colistin resistance,^41^ it probably provides cross-resistance between PHMB and colistin. Collectively, these data suggest that increased L-Ara4N modification of lipid A is a common mechanism for resistance to cationic antimicrobials in general, raising questions regarding cross-resistance between cationic disinfectants in healthcare and last-resort antibiotics such as polymyxins.

PHMB-adapted samples also showed a conserved D125G substitution in 2-keto-3-deoxygluconate (KDG) regulon repressor KdgR, which regulates KdgATK proteins responsible for KDG transportation and catabolism. Interestingly, a SNP causing D11G in KdgR was also found in all BAC-adapted samples (Table S1). Downstream expression of eda (KdgA) was lower in PHMB and BAC-tolerant samples, suggesting increased KdgR repressor activity. The exact impact this has on BAC and PHMB resistance is unclear.

### *K. pneumoniae* resistance to chlorocresol

Chlorocresol-adapted samples contained conserved mutations in *cpdA* and *yjcC* (Table 3), which regulate intracellular cAMP and c-di-GMP levels, respectively. The mutations resulted in S46G in CpdA and P248S in YjcC.

*yjcC* (synonymous *pdeC*) encodes a phosphodiesterase that hydrolyses c-di-GMP when dimerized,^42^ negatively regulating type 3 fimbriae expression and biofilm formation.^43^ The observed substitution is in the second transmembrane region, responsible for dimerization and protein activation.^42^ We hypothesize the substitution of this conserved proline^42^ impedes dimerization, increasing intracellular c-di-GMP, enhancing biofilm formation and type 3 fimbriae expression.

CpdA hydrolyses cAMP to 5′-adenosine monophosphate (AMP). Deletion of this protein causes intracellular cAMP to increase up to 4-fold,^44^ promoting biofilm formation in *K. pneumoniae* via type 3 fimbriae production.^45^ The adaptations may therefore increase biofilm formation, probably relating to mutations in fimbriae-related genes *htrE*, *fim_1* and NCTC13443_06216 (Table 3), which shares sequence homology with type 3 fimbria minor subunit MrkB.

Also conserved was a frameshift insertion in *marR*, truncating the *marRAB* operon repressor (T27N.*fs*X3) (Table 3). This protein is responsible for AMR-related changes in expression including down-regulation of OmpF, increased expression of AcrAB-TolC and resistance to oxidative stress via transcriptional activator SoxS.^46^ MarR truncation probably increases expression of AcrAB-TolC and SoxS, contributing to chlorocresol resistance through efflux and activation of the superoxide response regulon. However, downstream changes could not be confirmed by proteomics due to protein coverage limitations.

Interestingly, loss of MarR function has been linked to increased WaaY expression, responsible for phosphorylation of the inner core of LPS, increasing the net-negative charge of the bacterial outer surface and susceptibility to antimicrobial peptides.^47^ Therefore, truncation of MarR probably contributes to the collateral susceptibility of chlorocresol-adapted samples to BAC and DDAC observed previously,^10^ and potentially polymyxin B.

Chlorocresol-adapted samples also showed increased expression of the MdtC and BepD (MdtA) proteins (Figure 5, Table 4), all components of tripartite efflux complex MdtABC. This complex is responsible for resistance to novobiocin and detergent deoxycholate via TolC-dependent efflux,^48^ and is associated with resistance to multiple antibiotics in *K. pneumoniae* clinical strains.^49^ Therefore, this probably contributes to chlorocresol resistance.

**Table 4.** The 10 proteins that demonstrated the highest average increased expression change detected via label-free global proteomics analysis. Samples consisted of disinfectant-adapted *K. pneumoniae* NCTC 13443, and were compared with untreated parent samples. *n* = 3, except for BAC-adapted samples, where *n* = 5

Resistant samples	Expression change (log_2_ fold change)	Protein identifier	Full protein name
BAC	5.64	BudB	Acetolactate synthase
	5.64	ArnA	Bifunctional polymyxin resistance protein ArnA
	5.64	YfdX	YfdX-like protein
	5.06	NCTC13443_01223	Thioredoxin-like protein
	4.64	MalZ	Maltodextrin glucosidase
	4.32	FruB	Multiphosphoryl transfer protein
	3.84	FruB	Multiphosphoryl transfer protein
	3.64	AcrB	Efflux pump membrane transporter AcrB
	3.64	FrlD	Fructosamine kinase FrlD
	3.47	AcrA	Efflux pump membrane transporter AcrA
DDAC	5.64	YfdX	YfdX-like protein
	5.64	GlpK	Glycerol kinase
	5.64	BudC	Diacetyl reductase [(S)-acetoin forming]
	4.64	NCTC13443_03659	Putative NADH:flavin oxidoreductase
	4.06	FruB	Multiphosphoryl transfer protein
	4.06	ValS	Valine–tRNA ligase
	3.84	ScrY	Sucrose porin
	3.32	DmlA	D-malate dehydrogenase (decarboxylating)
	3.32	LysA	Diaminopimelate decarboxylase
	3.18	AldB	Alpha-acetolactate decarboxylase
PHMB	5.64	GlpK	Glycerol kinase
	5.64	NCTC13443_03659	Putative NADH:flavin oxidoreductase
	5.64	YfdX	YfdX-like protein
	5.64	NCTC13443_02382	Putative L-fucose isomerase, C-terminal
	5.64	NCTC13443_02379	Putative L-fucose isomerase, C-terminal
	5.64	BudC	Diacetyl reductase [(*S*)-acetoin forming]
	5.06	NCTC13443_01223	Thioredoxin-like protein
	5.06	SacA	Sucrose-6-phosphate hydrolase
	4.64	ThiC	Phosphomethylpyrimidine synthase
	4.64	NCTC13443_02381	Putative L-fucose isomerase, C-terminal
Chlorocresol	5.64	YebE	Inner membrane protein YebE
	5.64	MdtC	Multidrug resistance protein MdtC
	5.06	YfdX	YfdX-like protein
	5.06	BudC	Diacetyl reductase [(*S*)-acetoin forming]
	4.64	AldB	Alpha-acetolactate decarboxylase
	4.64	BepD	Multidrug resistance protein MdtA
	4.64	UspG	Universal stress protein G
	4.32	NCTC13443_03659	Putative NADH:flavin oxidoreductase
	4.06	HutU	Urocanate hydratase
	3.84	FdhF	Formate dehydrogenase
Bronopol	5.64	BudB	Acetolactate synthase
	5.64	YfdX	YfdX-like protein
	5.64	DDJ638005	—
	4.32	NCTC13443_03659	Putative NADH:flavin oxidoreductase
	4.06	NCTC13443_01223	Thioredoxin-like protein
	3.64	FruB	Multiphosphoryl transfer protein
	3.47	FrlD	Fructokinase
	3.47	FadB	Fatty acid oxidation complex subunit alpha
	3.32	NemA	*N*-ethylmaleimide reductase
	3.32	DkgB	2,5-didehydrogluconate reductase DkgB

It should be noted that sub-inhibitory concentrations of DMSO have been shown to alter gene expression,^50^ mitigate ROS damage^50^ and reduce biofilm formation in *P. aeruginosa*.^51^ As such, the specific impact of the presence of low DMSO concentrations on the observed adaptations remain unclear, particularly those associated with increased biofilm formation.

### *K. pneumoniae* resistance to bronopol

Bronopol-adapted samples carried conserved SNPs in transcriptional regulators *putA*, *rhaS* and *purR* (Table 3). *putA* and *rhaS_2* encode for regulators of the *put* and *cel* operons, respectively. PutA oxidizes proline and acts as an auto repressor of *putA* and *putP*. Increased PutA expression indicates the mutation impedes the repressor functionality.

The conserved R138L substitution in the purine biosynthesis (*pur*) operon repressor PurR is proximal to the *E. coli* PurR corepressor binding site. Nearby mutations can broaden corepressor binding specificity and enhance repression activity.^52^ As *purBCEHLM* were all significantly down-regulated (Figure 6), the mutation likely enhances PurR-mediated repression. This response has also been observed in *E. coli* exposed to oxidative and antibiotic stressors,^53^ although the specific mechanisms are unknown. As bronopol induces oxidative stress through the generation of ROS, these data support the hypothesis that PurR has a key regulatory role associated with oxidative stress response.

Bronopol-adapted samples also displayed conserved mutations in the secondary-messenger regulators *yjcC* and *cpdA*, alongside in fimbriae-associated *htrE*, *fim_1* and NCTC13443_06216 genes (Table 3). This suggests resistance through enhanced biofilm formation as seen with chlorocresol. Similarly, oxidative-stress-inducing hypochlorite and tellurite are associated with increased intracellular c-di-GMP levels in *P. aeruginosa*,^54,55^ alongside increased diguanylate cyclase activity,^54,55^ surface attachment^54^ and biofilm formation.^55^ This collectively highlights a link between intracellular c-di-GMP concentration, biofilm formation and bacterial resistance to oxidative stress-inducing antimicrobials such as bronopol.

Bronopol-adapted samples showed increased expression of thioredoxin-like protein NCTC13443_01223 and flavin oxidoreductase-like protein NCTC13443_03659 (Table 4). Thioredoxin proteins can reduce the disulphide bonds formed by the MOA of bronopol, mitigating bronopol-induced oxidative stress in adapted *K. pneumoniae* samples. As flavin oxidoreductase knockouts demonstrate high susceptibility to oxidative stress in *E. coli*^56^ and *Streptococcus pneumoniae*,^57^ increased expression probably has the opposite effect.

The flavin-dependant N-ethylmaleimide reductase NemA was among the top proteins up-regulated in bronopol-resistant samples (Table 4). This protein has previously been shown to be capable of breaking down electrophiles^58^ including 24,6-trinitrotoluene (TNT)^59^ in *E. coli*. As bronopol and TNT both contain nitro electrophilic groups, the significant up-regulation of NemA suggests a potential role in bronopol resistance via enzymatic degradation. The requirement of flavin cofactor explains the associated up-regulation of NADH:flavin oxidoreductase. The reduced expression of catalases KatE, KatG and superoxide dismutase SodB (Figure 6) supports the hypothesis that NemA can break down bronopol before it is able to form ROS.

### Conclusion

Chemical disinfectants are relied on worldwide across healthcare, industrial settings, the food sector and household environments. Understanding mechanisms that mitigate their efficacy is critical to combating the hundreds of millions of annual HAI cases. This molecular analysis of *K. pneumoniae* adaptation to common disinfectants provides novel insights into potential disinfectant resistance mechanisms of HAI-associated pathogens. The findings highlight similarities between mechanisms facilitating resistance to cationic antibiotics and disinfectants, raising questions about the risk of cross-resistance that can be expanded on in future work. Genetic variation between BAC and DDAC-adapted samples show how minor differences between similar agents can manifest distinct adaptations, as demonstrated by conserved efflux pump adaptations in DDAC-adapted samples that are not necessary for BAC resistance.

Chlorocresol and bronopol resistance has not been investigated previously. *K. pneumoniae* resistance to chlorocresol was associated with *marR* loss of function, increased MdtABC efflux complex expression and promoting biofilm formation, a mechanism shared with bronopol resistance alongside mitigation of cross-linking damage. Increased expression of N-ethylmaleimide reductase NemA may facilitate bronopol resistance via enzymatic degradation.

Collateral susceptibility of chlorocresol-resistant *K. pneumoniae* to QACs and cationic agents via MarR truncation highlights how adaptations to one antimicrobial often leaves organisms vulnerable to others, knowledge that can be used to improve efficacy of cleaning routines and infection control.

Future studies should validate the mechanisms implicated in this work. The impacts of QAC and PHMB adaptations associated with lipid A modification can be assessed via zeta potential analysis and plasmid-based complementation, while the extent of QAC-polymyxin cross-resistance can be confirmed through antimicrobial susceptibility assays. The role of NemA in bronopol resistance can be investigated through enzymatic activity assays.

This study identifies molecular mechanisms of disinfectant resistance in *K. pneumoniae* samples generated via stepwise adaptation, deepening our understanding of the potential routes that HAI pathogens can exploit to mitigate disinfectant efficacy.

## Supplementary Material

dlaf247_Supplementary_Data

## Repositories

Data has been deposited on NCBI under BioProject accession PRJNA1270664. Data are publicly available at: https://www.ncbi.nlm.nih.gov/bioproject/PRJNA1270664.

## Supplementary data

Figure S1 and Tables S1 to S3 are available as Supplementary data at *JAC-AMR* Online.
