# Supplementary material for: Molecular mechanisms of disinfectant resistance in Klebsiella pneumoniae
Source: JAC Antimicrob Resist. 2026 Jan 16;8(1):dlaf247. doi: 10.1093/jacamr/dlaf247 (PMC12809559; doi:10.1093/jacamr/dlaf247)
Supplement: dlaf247_Supplementary_Data [file dlaf247_supplementary_data.zip › MolMechanismsDisinfectantResKpneumoniae_Appendices_Revised.docx]

**APPENDIX TABLES AND FIGURES**

Table S1. Conserved mutations detected in *Klebsiella pneumoniae* NCTC 13443 benzalkonium chloride-adapted samples (n=5).

| **Gene** | **Variation Type** | **Gene annotation** | **Protein annotation** | **Protein Product** |
| --- | --- | --- | --- | --- |
| *ascG_4* | fs-del | 999delT | P333fs | LacI family transcriptional regulator |
| *cydA_5* | fs-del | 214delA | K72fs | Cytochrome bd2 |
| *entF_7* | fs-del | 1delA | M1fs | enterobactin synthase subunit F |
| *intA_3* | fs-del | 1161delA | K387fs | integrase family protein |
| NCTC13443_02922 | fs-del | 466delG | G156fs | SD repeat-containing cell surface protein |
| NCTC13443_03262 | fs-del | 110delG | R37fs | antibiotic biosynthesis monooxygenase |
| NCTC13443_04386 | fs-del | 679_680del | A227fs | gluconate 2-dehydrogenase subunit gamma |
| NCTC13443_05638 | fs-del | 84delT | F28fs | Uncharacterised protein |
| NCTC13443_06478 | fs-del | 142delG | D48fs | Tail fibre protein |
| *prlC* | fs-del | 1135delG | A379fs | oligopeptidase A |
| *puo* | fs-del | 537delC | F179fs | Putrescine oxidase |
| *traI_2* | fs-del | 435_436del | I145fs | conjugal transfer nickase/helicase TraI |
| *ada_2* | fs-ins | 165dupT | A56fs | ADA regulatory protein / Methylated-DNA-protein-cysteine methyltransferase |
| *bglH_2* | fs-ins | 148dupG | A50fs | maltoporin |
| *ccmE* | fs-ins | 191dupG | G64fs | cytochrome c-type biogenesis protein |
| *codB_1* | fs-ins | 394_395insA | A132fs | cytosine/purine/uracil/thiamine/allantoin permease family protein |
| *cstA_1* | fs-ins | 1604dupG | R535fs | Carbon starvation protein A |
| *cueR* | fs-ins | 22dupA | A7fs | HTH-type transcriptional regulator cueR |
| *dus_1* | fs-ins | 585dupC | Y195fs | tRNA dihydrouridine synthase A |
| *eamA* | fs-ins | 780dupA | I260fs | drug/metabolite transporter permease |
| *eutD* | fs-ins | 45dupC | R16fs | phosphate acetyltransferase |
| *gmuB_4* | fs-ins | 55dupA | M18fs | PTS system protein |
| *gmuD_2* | fs-ins | 90dupA | P31fs | beta-glucosidase |
| *livJ_1* | fs-ins | 403dupG | A135fs | hydrophobic amino acid ABC transporter periplasmic amino acid-binding protein |
| *moeB_2* | fs-ins | 448dupC | L150fs | molybdopterin biosynthesis protein MoeB |
| NCTC13443_02541 | fs-ins | 152dupT | L51fs | B12-dependent methionine synthase |
| NCTC13443_03143 | fs-ins | 110dupT | F37fs | putative enzyme |
| NCTC13443_03206 | fs-ins | 293dupT | V98fs | Uncharacterised protein |
| NCTC13443_05033 | fs-ins | 1704dupG | R569fs | thiamine biosynthesis protein ThiF |
| NCTC13443_05034 | fs-ins | 689dupG | G230fs | Uncharacterised protein |
| NCTC13443_05801 | fs-ins | 28dupT | T9fs | NAD(P)H-flavin oxidoreductase |
| NCTC13443_07163 | fs-ins | 119_120insGG | M40fs | diguanylate cyclase |
| *panF_3* | fs-ins | 346dupG | A115fs | sodium/panthothenate symporter |
| *qmcA* | fs-ins | 454dupG | V152fs | stomatin/prohibitin-family membrane protease subunit YbbK |
| *treF_2* | fs-ins | 797_798insGG | P266fs | cytoplasmic trehalase |
| *tyrP_1* | fs-ins | 552dupG | L184fs | tyrosine-specific transporter |
| *ves* | fs-ins | 394dupG | V132fs | Various environmental stresses-induced protein |
| *ycfS_2* | fs-ins | 268dupC | L90fs | LysM domain/ErfK/YbiS/YcfS/YnhG family protein |
| *yeaN_6* | fs-ins | 590dupC | S197fs | cyanate transport protein CynX |
| *yfcA* | fs-ins | 101dupG | G34fs | membrane protein YfcA |
| *mnmA* | nfs-del | 702_713del | 234_238del | thiouridylase |
| *malT* | nfs-del | 218_220del | 73_74del | transcriptional regulator MalT |
| acrB_6 | ns-SNP | G388A | E130K | RND efflux system |
| arnT | ns-SNP | A1370G | N457S | 4-amino-4-deoxy-L-arabinose transferase |
| aroF | ns-SNP | G61A | E21K | phospho-2-dehydro-3-deoxyheptonate aldolase |
| astB_1 | ns-SNP | C1226T | A409V | succinylarginine dihydrolase |
| atpA_3 | ns-SNP | G160A | G54S | ATP synthase subunit alpha |
| basS | ns-SNP | C203T | A68V | sensor protein BasS/PmrB |
| bioD_1 | ns-SNP | A334G | T112A | dithiobiotin synthetase |
| bsaA_2 | ns-SNP | C17T | P6L | glutathione peroxidase |
| btr | ns-SNP | C662T | S221L | 4-hydroxyphenylacetate catabolism regulatory protein HpaA |
| cmpB_2 | ns-SNP | A151G | T51A | nitrate ABC transporter |
| codB_1 | ns-SNP | G394C | A132P | cytosine/purine/uracil/thiamine/allantoin permease family protein |
| crp | ns-SNP | G363A | M121I | cyclic AMP receptor protein |
| cusC_1 | ns-SNP | A976G | T326A | copper/silver efflux system outer membrane protein CusC |
| cysA_2 | ns-SNP | C935T | P312L | sulfate and thiosulfate import ATP-binding protein CysA |
| cysK_1 | ns-SNP | T748C | F250L | cysteine synthase B |
| cysW_1 | ns-SNP | C1307T | P436L | ABC transporter membrane protein |
| cytR_3 | ns-SNP | C129A | D43E | sugar-binding domain protein |
| dadX | ns-SNP | G73A | A25T | alanine racemase |
| dam_1 | ns-SNP | A230G | N77S | methyl-directed repair DNA adenine methylase |
| degP_1 | ns-SNP | A833G | N278S | HtrA protease/chaperone protein |
| deoC_1 | ns-SNP | C461T | A154V | deoxyribose-phosphate aldolase |
| dmlA_1 | ns-SNP | A131G | E44G | tartrate dehydrogenase |
| dppA_5 | ns-SNP | G79A | D27N | antimicrobial peptide ABC transporter substrate-binding protein SapA |
| entF_4 | ns-SNP | C262T | R88C | enterobactin synthase subunit F |
| envZ_2 | ns-SNP | G559A | V187M | Osmolarity sensory histidine kinase EnvZ |
| eptA_1 | ns-SNP | T913C | F305L | putative cell division protein |
| fabF_1 | ns-SNP | A1111G | T371A | 3-oxoacyl-(acyl carrier protein) synthase II |
| fepB | ns-SNP | G490A | G164S | Ferric enterobactin-binding periplasmic protein FepB |
| fimD_9 | ns-SNP | G1240A | A414T | outer membrane protein for export and assembly of type 1 fimbriae |
| fimD_9 | ns-SNP | G311A | G104D | outer membrane protein for export and assembly of type 1 fimbriae |
| fis | ns-SNP | G166A | A56T | DNA-binding protein Fis |
| ganB | ns-SNP | C872T | A291V | galactosidase |
| gbh | ns-SNP | C565T | R189C | Agmatinase |
| gcvA_2 | ns-SNP | G310A | G104S | glycine cleavage system transcriptional activator |
| glnE | ns-SNP | G94A | A32T | glutamate-ammonia-ligase adenylyltransferase |
| gloA | ns-SNP | G89A | R30H | Lactoylglutathione lyase |
| glyA | ns-SNP | C890T | A297V | glycine hydroxymethyltransferase |
| gsiA_9 | ns-SNP | G302A | C101Y | peptide transport system ATP-binding protein SapD |
| hcpA_3 | ns-SNP | G70A | A24T | Hcp family type VI secretion system effector |
| icaB | ns-SNP | C958T | R320W | polysaccharide deacetylase |
| idi_2 | ns-SNP | G73A | E25K | isopentenyl-diphosphate delta-isomerase |
| ilvD_3 | ns-SNP | G676A | E226K | phosphogluconate dehydratase |
| kdgR | ns-SNP | A32G | D11G | transcriptional regulator KdgR |
| lacE_1 | ns-SNP | T151C | F51L | PTS system protein |
| lamB_4 | ns-SNP | C1246T | P416S | maltoporin |
| leuA_3 | ns-SNP | G806A | G269D | 2-isopropylmalate synthase |
| lolE | ns-SNP | T493C | W165R | outer membrane-specific lipoprotein transporter subunit LolE |
| lon_2 | ns-SNP | G662A | G221D | DNA-binding ATP-dependent protease La |
| lptB_2 | ns-SNP | T158C | V53A | lipopolysaccharide ABC transporter |
| luxS | ns-SNP | G169A | E57K | S-ribosylhomocysteine lyase |
| lysP_1 | ns-SNP | T449C | F150S | Lysine-specific permease |
| malH | ns-SNP | G56A | G19E | maltose-6'-phosphate glucosidase |
| mipA | ns-SNP | G97T | G33W | MltA-interacting protein MipA |
| msbA_2 | ns-SNP | A122G | D41G | lipid A export ATP-binding/permease MsbA |
| murG_2 | ns-SNP | G424A | V142I | UDP-N-acetylglucosamine-N-acetylmuramyl-(pentapeptide) pyrophosphoryl-undecaprenol N-acetylglucosamine transferase |
| mutY | ns-SNP | G397A | G133S | adenine DNA glycosylase |
| NCTC13443_00226 | ns-SNP | C869T | S290F | Uncharacterised protein |
| NCTC13443_00323 | ns-SNP | A221G | Y74C | lipoprotein |
| NCTC13443_01284 | ns-SNP | G424A | G142R | 5-keto-2-deoxygluconokinase |
| NCTC13443_01360 | ns-SNP | G958A | A320T | amine oxidase, flavin-containing |
| NCTC13443_01431 | ns-SNP | A620G | D207G | transposase |
| NCTC13443_01522 | ns-SNP | C623T | T208I | phospholipase, patatin family |
| NCTC13443_01557 | ns-SNP | G152A | C51Y | inner membrane protein CreD |
| NCTC13443_02085 | ns-SNP | C71T | A24V | fumarate reductase/succinate dehydrogenase flavoprotein domain-containing protein |
| NCTC13443_02244 | ns-SNP | G29A | G10D | Uncharacterised protein |
| NCTC13443_02252 | ns-SNP | C68T | T23M | Uncharacterised protein |
| NCTC13443_02928 | ns-SNP | C164T | A55V | glycosyl transferase |
| NCTC13443_03209 | ns-SNP | G928A | G310S | terminase, ATPase subunit |
| NCTC13443_03672 | ns-SNP | G634A | A212T | auxin efflux carrier |
| NCTC13443_03977 | ns-SNP | A236G | Y79C | AraC family transcriptional regulator |
| NCTC13443_03985 | ns-SNP | G35A | C12Y | GntR family transcriptional regulator |
| NCTC13443_04134 | ns-SNP | T1336C | Y446H | alpha-L-rhamnosidase |
| NCTC13443_04491 | ns-SNP | C698T | A233V | 3-beta hydroxysteroid dehydrogenase/isomerase family protein |
| NCTC13443_04522 | ns-SNP | C1121T | A374V | selenoprotein O-like protein |
| NCTC13443_04782 | ns-SNP | C89T | A30V | phosphogluconate dehydratase |
| NCTC13443_04839 | ns-SNP | T769C | Y257H | gp9 |
| NCTC13443_04849 | ns-SNP | A352G | T118A | Lysozyme |
| NCTC13443_04850 | ns-SNP | G244A | G82S | Uncharacterised protein |
| NCTC13443_04875 | ns-SNP | G226A | E76K | TolA protein |
| NCTC13443_04971 | ns-SNP | A575G | H192R | membrane protein |
| NCTC13443_05458 | ns-SNP | A139G | I47V | Uncharacterised protein |
| NCTC13443_05650 | ns-SNP | A478G | T160A | 2-polyprenylphenol hydroxylase related flavodoxin oxidoreductase |
| NCTC13443_06074 | ns-SNP | C224T | A75V | ImpA family type VI secretion-associated protein |
| NCTC13443_06079 | ns-SNP | A190G | T64A | Uncharacterised protein |
| NCTC13443_06203 | ns-SNP | A247G | S83G | Oligogalacturonate lyase |
| NCTC13443_06478 | ns-SNP | A482G | Y161C | Tail fiber protein |
| NCTC13443_06601 | ns-SNP | A101C | D34A | hemolysin |
| NCTC13443_06709 | ns-SNP | G193A | V65I | putative cation transporter |
| NCTC13443_06725 | ns-SNP | A1664C | N555T | membrane protein |
| NCTC13443_06725 | ns-SNP | G1376A | G459E | membrane protein |
| NCTC13443_07298 | ns-SNP | A232G | S78G | type-F conjugative transfer system mating-pair stabilization protein TraN |
| NCTC13443_07417 | ns-SNP | C248T | A83V | StrB |
| nfdA_1 | ns-SNP | G1453A | A485T | exoenzymes regulatory protein AepA |
| nfnB_1 | ns-SNP | G443A | R148H | Oxygen-insensitive NAD(P)H nitroreductase |
| nikA_2 | ns-SNP | A368G | Q123R | nickel ABC transporter, periplasmic nickel-binding protein NikA |
| norG | ns-SNP | G47A | G16D | GntR family transcriptional regulator |
| nuoM_1 | ns-SNP | C212T | P71L | NADH-ubiquinone oxidoreductase subunit M |
| phoC_2 | ns-SNP | T442C | S148P | acid phosphatase |
| phoR_4 | ns-SNP | A1046G | E349G | osmosensitive K+ channel histidine kinase KdpD |
| pldA | ns-SNP | C17T | A6V | phospholipase A1 |
| plsB | ns-SNP | G1714A | A572T | glycerol-3-phosphate acyltransferase |
| plsC | ns-SNP | T433C | F145L | 1-acyl-sn-glycerol-3-phosphate acyltransferase |
| ppsA_1 | ns-SNP | G179A | G60D | phosphoenolpyruvate synthase |
| priC | ns-SNP | A121G | T41A | primosomal replication protein N'' |
| pulB | ns-SNP | C257T | A86V | pullulanase-specific type II secretion system component B |
| rhaS_3 | ns-SNP | A203G | D68G | AraC family transcriptional regulator |
| rhlE | ns-SNP | A754G | T252A | ATP-dependent RNA helicase RhlE |
| rluE | ns-SNP | A550G | T184A | ribosomal large subunit pseudouridine synthase E |
| rnfG | ns-SNP | G289A | A97T | electron transport complex protein RnfG |
| rplN | ns-SNP | G136A | A46T | 50S ribosomal protein L14 |
| rpsD_1 | ns-SNP | T77C | V26A | 30S ribosomal protein S4 |
| rutG | ns-SNP | G1183A | G395S | Uracil permease |
| sbcB | ns-SNP | G1090A | A364T | exodeoxyribonuclease I |
| sbcD_2 | ns-SNP | T19C | S7P | exonuclease SbcD |
| setB | ns-SNP | G218A | G73D | sugar efflux transporter B |
| setB | ns-SNP | G775A | G259S | sugar efflux transporter B |
| surA | ns-SNP | G1015A | G339S | survival protein SurA precursor (Peptidyl-prolyl cis-trans isomerase SurA) |
| surE_1 | ns-SNP | C302T | S101F | stationary phase survival protein SurE |
| thiB | ns-SNP | A418G | S140G | thiamin ABC transporter |
| tktA_1 | ns-SNP | A845G | D282G | transketolase |
| tonB | ns-SNP | A206G | E69G | transporter |
| trpE_1 | ns-SNP | G526A | A176T | anthranilate synthase |
| tusE | ns-SNP | T137C | V46A | tRNA 2-thiouridine synthesizing protein E |
| tvaI | ns-SNP | C770T | P257L | maltodextrin glucosidase |
| ybhS_2 | ns-SNP | A664G | T222A | ABC transporter |
| ydcR_1 | ns-SNP | G664A | E222K | GntR family transcriptional regulator |
| yejB | ns-SNP | G787A | E263K | Oligopeptide transport system permease OppB |
| ygbM_2 | ns-SNP | G193A | D65N | hydroxypyruvate isomerase |
| yggG_1 | ns-SNP | G1405A | A469T | exported zinc metalloprotease YfgC |
| afr_1 | stop gain | G488A | W163X | Myo-inositol 2-dehydrogenase |
| narX_2 | stop gain | G294A | W98X | nitrate/nitrite sensor protein |
| ssuB_5 | stop gain | C211T | Q71X | alkanesulfonates ABC transporter ATP-binding protein / Sulfonate ABC transporter |

*fs-del: frameshift deletion. fs-ins: frameshift insertion. nfs-del: non-frameshift deletion. ns-SNP: non-synonymous single nucleotide polymorphism.*

Table S2. Conserved mutations detected in *Klebsiella pneumoniae* NCTC 13443 didecyldimethylammonium chloride-adapted samples (n=3).

| **Gene** | **Variation Type** | **Gene annotation** | **Protein annotation** | **Protein Product** |
| --- | --- | --- | --- | --- |
| ampE | fs-del | 459delG | V153fs | AmpE protein |
| artM_1 | fs-del | 400delA | S134fs | phosphate transport ATP-binding protein PstB |
| fusA_1 | fs-del | 269delC | P90fs | translation elongation factor G |
| galS | fs-del | 919delG | A307fs | Mgl repressor and galactose ultrainduction factor GalS |
| NCTC13443_02326 | fs-del | 99delG | Q33fs | outer membrane protein romA |
| NCTC13443_04371 | fs-del | 99delG | E33fs | Uncharacterised protein |
| NCTC13443_04880 | fs-del | 114delC | A38fs | Uncharacterised protein |
| NCTC13443_05638 | fs-del | 84delT | F28fs | Uncharacterised protein |
| NCTC13443_07068 | fs-del | 328delG | G110fs | conserved hypothetical signal peptide protein |
| prlC | fs-del | 1135delG | A379fs | oligopeptidase A |
| tetA_2 | fs-del | 177delG | G59fs | multidrug-efflux transporter, major facilitator superfamily (MFS) |
| traI_3 | fs-del | 489delG | Q163fs | conjugal transfer nickase/helicase TraI |
| ydcR_2 | fs-del | 789delA | K263fs | GntR family transcriptional regulator |
| yjcC_2 | fs-del | 356delC | P119fs | cyclic diguanylate phosphodiesterase (EAL) domain-containing protein |
| ada_2 | fs-ins | 165dupT | A56fs | ADA regulatory protein / Methylated-DNA-protein-cysteine methyltransferase |
| artI | fs-ins | 405dupA | V136fs | arginine ABC transporter substrate-binding protein |
| bglH_2 | fs-ins | 148dupG | A50fs | maltoporin |
| copA_5 | fs-ins | 1072_1073insCC | L358fs | copper resistance protein A |
| copA_5 | fs-ins | 651dupG | L218fs | copper resistance protein A |
| ctfA | fs-ins | 606dupC | G203fs | bifunctional putative acetyl-CoA:acetoacetyl-CoA transferase: alpha subunit/beta subunit |
| feaB_1 | fs-ins | 477dupG | L159fs | aldehyde dehydrogenase |
| gmuB_4 | fs-ins | 55dupA | M18fs | PTS system protein |
| mhpC | fs-ins | 420_421insGG | T141fs | 2-hydroxy-6-ketonona-2,4-dienedioic acid hydrolase |
| moeB_2 | fs-ins | 448dupC | L150fs | molybdopterin biosynthesis protein MoeB |
| narZ | fs-ins | 673dupC | L224fs | respiratory nitrate reductase subunit alpha |
| NCTC13443_01315 | fs-ins | 29dupT | L10fs | Uncharacterised protein |
| NCTC13443_03229 | fs-ins | 2106dupT | T703fs | phage protein |
| NCTC13443_04866 | fs-ins | 756dupG | K253fs | Origin specific replication binding factor |
| NCTC13443_05427 | fs-ins | 1446dupG | Q483fs | putative kinase |
| NCTC13443_06493 | fs-ins | 51_52insT | A18fs | terminase, endonuclease subunit (GpM) |
| nepI_3 | fs-ins | 389_390insGG | L130fs | putative MFS-family transport protein |
| nikC_2 | fs-ins | 419dupG | W140fs | nickel transport system permease protein NikC |
| qseF | fs-ins | 301dupG | V101fs | sensory histidine kinase YfhA |
| repB | fs-ins | 856dupA | D285fs | DNA replication |
| treF_2 | fs-ins | 797_798insGG | P266fs | cytoplasmic trehalase |
| uppP | fs-ins | 529dupG | A177fs | undecaprenyl pyrophosphate phosphatase |
| ycfS_2 | fs-ins | 268dupC | L90fs | LysM domain/ErfK/YbiS/YcfS/YnhG family protein |
| malT | nfs-del | 702_713del | 234_238del | transcriptional regulator MalT |
| mnmA | nfs-del | 218_220del | 73_74del | thiouridylase |
| NCTC13443_07015 | nfs-del | 181_198del | 61_66del | Uncharacterised protein |
| traD_1 | nfs-ins | 1849_1850ins  AACAGCCAC | E617delins  EQPQ | conjugal transfer protein TraD |
| acrB_6 | ns-SNP | G388A | E130K | RND efflux system |
| acrR_2 | ns-SNP | T242C | F81S | multidrug efflux pump acrAB operon transcription repressor |
| acs_2 | ns-SNP | T479C | L160P | acetyl-CoA synthetase |
| afr_2 | ns-SNP | C143T | A48V | Myo-inositol 2-dehydrogenase |
| ampH | ns-SNP | T673C | S225P | penicillin-binding protein AmpH |
| ansA_2 | ns-SNP | G86A | G29D | L-asparaginase |
| araC_3 | ns-SNP | G4A | A2T | Arabinose operon regulatory protein |
| aroE_6 | ns-SNP | C34T | H12Y | shikimate 5-dehydrogenase |
| artI | ns-SNP | T32C | V11A | arginine ABC transporter substrate-binding protein |
| basS | ns-SNP | C203T | A68V | sensor protein BasS/PmrB |
| bepE_3 | ns-SNP | T1058C | V353A | RND multidrug efflux transporter, Acriflavin resistance protein |
| bglK | ns-SNP | T665C | L222P | N-acetylmannosamine kinase |
| bioD_1 | ns-SNP | A334G | T112A | dithiobiotin synthetase |
| cadA | ns-SNP | C454T | H152Y | lysine decarboxylase 1 |
| cbiA | ns-SNP | C389T | S130F | cobyrinic acid a,c-diamide synthase |
| cydD | ns-SNP | A1652G | D551G | transport ATP-binding protein CydD |
| cysA_2 | ns-SNP | C935T | P312L | sulfate and thiosulfate import ATP-binding protein CysA |
| cytR_3 | ns-SNP | C129A | D43E | sugar-binding domain protein |
| dam_3 | ns-SNP | C76T | P26S | DNA adenine methylase |
| ddrA_3 | ns-SNP | T181C | S61P | propanediol dehydratase reactivation factor large subunit |
| dmsA_1 | ns-SNP | T377C | L126P | biotin sulfoxide reductase |
| eptA_1 | ns-SNP | T913C | F305L | putative cell division protein |
| fabF_1 | ns-SNP | A1111G | T371A | 3-oxoacyl-(acyl carrier protein) synthase II |
| fepB | ns-SNP | G490A | G164S | Ferric enterobactin-binding periplasmic protein FepB |
| fimA_3 | ns-SNP | C503T | A168V | fimbrial protein |
| fimD_9 | ns-SNP | G1240A | A414T | outer membrane protein for export and assembly of type 1 fimbriae |
| fimD_9 | ns-SNP | T1630C | Y544H | outer membrane protein for export and assembly of type 1 fimbriae |
| fsaB | ns-SNP | G22A | A8T | fructose-6-phosphate aldolase |
| fucO_1 | ns-SNP | C431T | P144L | Lactaldehyde reductase |
| galM | ns-SNP | G413A | G138D | aldose 1-epimerase |
| gcvA_8 | ns-SNP | A635G | N212S | glycine cleavage system transcriptional activator |
| glpQ_3 | ns-SNP | C359T | P120L | glycerophosphoryl diester phosphodiesterase |
| gltA_2 | ns-SNP | C278T | S93F | Citrate synthase (si) |
| gltR_2 | ns-SNP | C761T | T254I | LysR family transcriptional regulator |
| gmk | ns-SNP | C346T | P116S | guanylate kinase |
| gntU_2 | ns-SNP | A262G | I88V | Low-affinity gluconate/H+ symporter GntU |
| gsiA_17 | ns-SNP | G556A | A186T | ABC transporter ATP-binding protein |
| gsk_1 | ns-SNP | C596T | A199V | inosine-guanosine kinase |
| hflB_2 | ns-SNP | A173G | E58G | ATP-dependent metalloprotease |
| hflX_1 | ns-SNP | T243A | D81E | GTP-binding protein HflX |
| hmuT_2 | ns-SNP | G197A | S66N | Periplasmic hemin-binding protein |
| hrsA | ns-SNP | T17C | V6A | PTS system transporter subunit IIA |
| hsdM_5 | ns-SNP | A908G | H303R | type I restriction-modification system, M subunit |
| hypD | ns-SNP | G851A | G284D | [NiFe] hydrogenase metallocenter assembly protein HypD |
| hypF | ns-SNP | C1540T | R514W | hydrogenase metallocenter assembly protein HypF |
| idi_2 | ns-SNP | G73A | E25K | isopentenyl-diphosphate delta-isomerase |
| ileS | ns-SNP | A1484G | H495R | isoleucyl-tRNA synthetase |
| infB_1 | ns-SNP | A1607G | N536S | translation initiation factor 2 |
| kdgT | ns-SNP | A121G | T41A | 2-keto-3-deoxygluconate permease |
| kefA_3 | ns-SNP | T302C | L101P | potassium efflux system KefA protein / Small-conductance mechanosensitive channel |
| ksdD_2 | ns-SNP | G949A | A317T | FAD-dependent oxidoreductase |
| lacE_1 | ns-SNP | T151C | F51L | PTS system protein |
| lipA | ns-SNP | T707C | V236A | lipoate synthase |
| livF_1 | ns-SNP | T257C | V86A | branched chain amino acid ABC transporter ATPase |
| lptG | ns-SNP | G893A | G298D | Permease |
| ltrA_5 | ns-SNP | C1484T | A495V | Retron-type reverse transcriptase |
| ltrA_7 | ns-SNP | C1462T | H488Y | Retron-type reverse transcriptase |
| map_1 | ns-SNP | T400C | Y134H | methionine aminopeptidase |
| mazG | ns-SNP | A350G | Q117R | Nucleoside triphosphate pyrophosphohydrolase MazG |
| mdtC_1 | ns-SNP | G3015A | M1005I | multidrug transporter MdtB |
| metI_4 | ns-SNP | A466G | T156A | methionine ABC transporter permease |
| metQ_2 | ns-SNP | G97A | G33S | methionine ABC transporter substrate-binding protein |
| mipA | ns-SNP | G97T | G33W | MltA-interacting protein MipA |
| moeB_2 | ns-SNP | G30A | M10I | molybdopterin biosynthesis protein MoeB |
| msbA_2 | ns-SNP | A122G | D41G | lipid A export ATP-binding/permease MsbA |
| mutY | ns-SNP | G397A | G133S | adenine DNA glycosylase |
| narX_1 | ns-SNP | G280A | G94S | nitrate/nitrite sensor protein |
| narZ | ns-SNP | T2044C | F682L | respiratory nitrate reductase subunit alpha |
| NCTC13443_00226 | ns-SNP | C869T | S290F | Uncharacterised protein |
| NCTC13443_00262 | ns-SNP | C110T | T37I | AsmA family protein |
| NCTC13443_00263 | ns-SNP | G457A | D153N | AsmA family protein |
| NCTC13443_00552 | ns-SNP | T55C | W19R | Protein of uncharacterised function (DUF3748) |
| NCTC13443_01208 | ns-SNP | A439G | S147G | surface antigen |
| NCTC13443_01284 | ns-SNP | G424A | G142R | 5-keto-2-deoxygluconokinase |
| NCTC13443_01516 | ns-SNP | A5G | N2S | ribosomal-protein-S18p-alanine acetyltransferase |
| NCTC13443_01559 | ns-SNP | T221C | L74P | TrmH family RNA methyltransferase |
| NCTC13443_01882 | ns-SNP | T427C | C143R | transpeptidase |
| NCTC13443_02252 | ns-SNP | C518T | P173L | Uncharacterised protein |
| NCTC13443_02429 | ns-SNP | C757T | P253S | Uncharacterized conserved protein |
| NCTC13443_02904 | ns-SNP | T1751C | L584P | ABC transporter ATPase |
| NCTC13443_02920 | ns-SNP | A3422G | D1141G | SD repeat-containing cell surface protein |
| NCTC13443_02923 | ns-SNP | A187G | T63A | SD repeat-containing cell surface protein |
| NCTC13443_03204 | ns-SNP | A575G | D192G | Predicted ATP-binding protein involved in virulence |
| NCTC13443_03571 | ns-SNP | C830T | A277V | ATP-dependent helicase hrpA |
| NCTC13443_03769 | ns-SNP | G10A | E4K | inner membrane protein |
| NCTC13443_03783 | ns-SNP | C100T | H34Y | Uncharacterised protein |
| NCTC13443_03908 | ns-SNP | G472A | A158T | alpha/beta hydrolase |
| NCTC13443_04116 | ns-SNP | T106C | F36L | transcriptional regulator TetR family |
| NCTC13443_04134 | ns-SNP | T1336C | Y446H | alpha-L-rhamnosidase |
| NCTC13443_04134 | ns-SNP | G304A | A102T | alpha-L-rhamnosidase |
| NCTC13443_04491 | ns-SNP | C698T | A233V | 3-beta hydroxysteroid dehydrogenase/isomerase family protein |
| NCTC13443_04522 | ns-SNP | C1121T | A374V | selenoprotein O-like protein |
| NCTC13443_04782 | ns-SNP | C89T | A30V | phosphogluconate dehydratase |
| NCTC13443_04838 | ns-SNP | C152T | A51V | Uncharacterised protein |
| NCTC13443_04875 | ns-SNP | G490A | E164K | TolA protein |
| NCTC13443_04995 | ns-SNP | A245G | D82G | Uncharacterised protein |
| NCTC13443_04995 | ns-SNP | G157A | A53T | Uncharacterised protein |
| NCTC13443_05221 | ns-SNP | C55T | R19C | methyltransferase |
| NCTC13443_05444 | ns-SNP | C1148T | T383I | phage portal protein |
| NCTC13443_05683 | ns-SNP | T233C | I78T | Uncharacterised protein |
| NCTC13443_05752 | ns-SNP | G1930A | A644T | protein acetyltransferase |
| NCTC13443_06074 | ns-SNP | C224T | A75V | ImpA family type VI secretion-associated protein |
| NCTC13443_06084 | ns-SNP | G43A | V15I | ImcF domain-containing protein |
| NCTC13443_06163 | ns-SNP | A424G | T142A | prepilin peptidase dependent protein B |
| NCTC13443_06478 | ns-SNP | A482G | Y161C | Tail fiber protein |
| NCTC13443_06493 | ns-SNP | G51T | Q17H | terminase, endonuclease subunit (GpM) |
| NCTC13443_06601 | ns-SNP | A101C | D34A | hemolysin |
| NCTC13443_06709 | ns-SNP | G193A | V65I | putative cation transporter |
| NCTC13443_06725 | ns-SNP | A1664C | N555T | membrane protein |
| NCTC13443_06725 | ns-SNP | G1376A | G459E | membrane protein |
| NCTC13443_06845 | ns-SNP | G730A | V244I | YccS/YhfK family integral membrane protein |
| NCTC13443_06939 | ns-SNP | G388A | V130M | branched-chain amino acid transport system permease LivM |
| NCTC13443_07086 | ns-SNP | A89G | N30S | transposase |
| NCTC13443_07459 | ns-SNP | T1472A | L491H | transposase |
| nrdA_4 | ns-SNP | G217A | G73S | ribonucleotide reductase of class Ia |
| nrdF_1 | ns-SNP | A215G | H72R | ribonucleotide-diphosphate reductase subunit beta |
| nuoN | ns-SNP | G418A | A140T | NADH-ubiquinone oxidoreductase subunit N |
| oxyR_2 | ns-SNP | A20G | K7R | LysR family transcriptional regulator |
| pepN | ns-SNP | A2381G | N794S | membrane alanine aminopeptidase N |
| pflA_2 | ns-SNP | A737G | Y246C | pyruvate formate-lyase activating enzyme |
| phnD_1 | ns-SNP | G205A | A69T | phosphonate ABC transporter substrate-binding protein |
| pitA_1 | ns-SNP | T266C | L89P | Low-affinity inorganic phosphate transporter |
| pkn1_2 | ns-SNP | C643T | L215F | serine/threonine kinase |
| pldA | ns-SNP | C17T | A6V | phospholipase A1 |
| plsB | ns-SNP | G1714A | A572T | glycerol-3-phosphate acyltransferase |
| plsC | ns-SNP | T433C | F145L | 1-acyl-sn-glycerol-3-phosphate acyltransferase |
| potA_3 | ns-SNP | T758C | V253A | ABC transporter |
| ppsE | ns-SNP | T857A | V286E | irp1 |
| pqiB | ns-SNP | A1454G | N485S | paraquat-inducible protein B |
| proW_1 | ns-SNP | T172C | F58L | L-proline glycine betaine ABC transport system permease protein ProW |
| pstC | ns-SNP | C767T | A256V | phosphate ABC transporter permease |
| purF | ns-SNP | G632A | S211N | Amidophosphoribosyltransferase |
| ratB | ns-SNP | A220G | I74V | yfjF |
| rihA | ns-SNP | G508A | D170N | ribonucleoside hydrolase 1 |
| rna_1 | ns-SNP | G290A | G97D | ribonuclease I |
| rnfG | ns-SNP | G289A | A97T | electron transport complex protein RnfG |
| rplN | ns-SNP | G136A | A46T | 50S ribosomal protein L14 |
| serC | ns-SNP | A100G | T34A | phosphoserine aminotransferase |
| setB | ns-SNP | G218A | G73D | sugar efflux transporter B |
| soxS_3 | ns-SNP | T275C | V92A | transcriptional activator RamA |
| soxS_3 | ns-SNP | C298T | P100S | transcriptional activator RamA |
| sra | ns-SNP | G82A | D28N | 30S ribosomal subunit S22 |
| ssuA_3 | ns-SNP | C830T | A277V | nitrate/sulfonate/bicarbonate ABC transporter periplasmic protein |
| surA | ns-SNP | G1015A | G339S | survival protein SurA precursor (Peptidyl-prolyl cis-trans isomerase SurA) |
| surE_1 | ns-SNP | C302T | S101F | stationary phase survival protein SurE |
| tcyC_4 | ns-SNP | C218T | A73V | glutamine ABC transporter ATP-binding protein |
| thiI | ns-SNP | C61T | R21C | thiamine biosynthesis protein thiI |
| thiK_2 | ns-SNP | C187T | P63S | thiamine kinase |
| tktB_4 | ns-SNP | C214T | P72S | transketolase |
| tnsB_3 | ns-SNP | A535G | T179A | Transposon Tn7 transposition protein tnsB |
| tonB | ns-SNP | A206G | E69G | transporter |
| trbB_1 | ns-SNP | A505G | T169A | conjugal transfer protein TrbB |
| trmJ_3 | ns-SNP | T356C | V119A | tRNA:Cm32/Um32 methyltransferase |
| ugpQ_2 | ns-SNP | A572G | N191S | glycerophosphoryl diester phosphodiesterase |
| uvrY | ns-SNP | A535G | K179E | BarA-associated response regulator UvrY (GacA, SirA) |
| uxuA_1 | ns-SNP | A331G | T111A | mannonate dehydratase |
| vanB | ns-SNP | G466A | A156T | vanillate O-demethylase oxidoreductase |
| yabI_2 | ns-SNP | G127A | G43S | DedA-family integral membrane protein |
| ybhE | ns-SNP | C280T | R94C | 6-phosphogluconolactonase |
| yccS | ns-SNP | C277T | L93F | efflux (PET) family inner membrane protein YccS |
| ycgG_7 | ns-SNP | T169C | W57R | fimbrial protein |
| ydcR_3 | ns-SNP | G277A | G93S | transcriptional regulator of pyridoxine metabolism |
| ydcU_2 | ns-SNP | G208A | G70S | Ferric iron ABC transporter |
| yedY_1 | ns-SNP | G72A | M24I | sulfite oxidase subunit YedY |
| yejB | ns-SNP | G787A | E263K | Oligopeptide transport system permease OppB |
| yesR | ns-SNP | A196G | T66A | Rhamnogalacturonides degradation protein RhiN |
| yhdH | ns-SNP | G535A | V179M | quinone oxidoreductase |
| yhhX | ns-SNP | C409A | L137M | putative dehydrogenase |
| yhjK | ns-SNP | C1589T | T530I | protein yhjK |
| yicL | ns-SNP | G214A | A72T | inner membrane transporter yicL |
| yqiC | ns-SNP | T154C | F52L | Uncharacterized protein conserved in bacteria |
| ywnH | ns-SNP | C401T | A134V | N-acetyltransferase-like protein |
| yxeN | ns-SNP | C374T | A125V | glutamine ABC transporter ATP-binding protein |
| yycG | ns-SNP | G481A | A161T | histidine kinase |
| fucK_2 | stop gain | G612A | W204X | L-fuculokinase |
| narX_2 | stop gain | G294A | W98X | nitrate/nitrite sensor protein |
| NCTC13443_02172 | stop gain | G743A | W248X | transposase |
| NCTC13443_03443 | stop gain | C1093T | Q365X | OpgC protein |
| NCTC13443_03481 | stop gain | C88T | Q30X | type VI secretion protein |
| NCTC13443_03908 | stop gain | G815A | W272X | alpha/beta hydrolase |
| ydiM | stop gain | C1168T | Q390X | MFS family transporter |
| nadR_2 | stop loss | A1031G | X344W | NadR transcriptional regulator / Nicotinamide-nucleotide adenylyltransferase |
| NCTC13443_07404 | stop loss | 431delA | X144 | antirestriction protein |
| scoA | stop loss | T706C | X236R | succinyl-CoA:3-ketoacid-coenzyme A transferase subunitA |

*fs-del: frameshift deletion. fs-ins: frameshift insertion. nfs-del: non-frameshift deletion. nfs-ins: non-frameshift insertion. ns-SNP: non-synonymous single nucleotide polymorphism.*

Table S3. Conserved mutations detected in antimicrobial resistance genes in *Klebsiella pneumoniae* NCTC 13443 disinfectant-adapted samples. Antimicrobial resistance genes detected via Antimicrobial Resistance Finder plus programme. Mutation distribution: 1 = resistant samples that carried the mutation.

| **Gene** | **Protein product** | **Mutation** | **Mutation Type** | **Ref. Seq.** | **Alt. Seq.** | **Mutation distribution** | | | | |
| --- | --- | --- | --- | --- | --- | --- | --- | --- | --- | --- |
|  |  |  |  |  |  | **BAC** | **DDAC** | **PHMB** | **CC** | **BR** |
| *basS* | Sensor protein BasS/PmrB | A68V | non-synonymous SNP | G | A | 1 | 1 |  |  |  |
| *basS* | Sensor protein BasS/PmrB | T157P | non-synonymous SNP | T | G |  |  | 1 |  |  |
| *bepE_3* | RND multidrug efflux transporter | V353A | non-synonymous SNP | T | C |  | 1 |  |  |  |
| *copA_5* | Copper resistance protein A | L358R.fsX7 | frameshift insertion | - | GG |  | 1 |  |  |  |
| *copA_5* | Copper resistance protein A | L218P.fsX12 | frameshift insertion | - | C |  | 1 |  |  |  |
| NCTC13443_07417 | 3'-kinase StrB | A83V | non-synonymous SNP | C | T | 1 |  |  |  |  |

*BAC: benzalkonium chloride-adapted sample. DDAC: didecyldimethylammonium chloride-adapted sample. PHMB: polyhexamethylene biguanide-adapted sample. CC: chlorocresol-adapted sample. BR: bronopol-adapted sample. SNP: single nucleotide polymorphism. RND: resistance-nodulation-division.*

Figure S1. Heatmaps showing gene ontology (GO) enrichment analysis of *Klebsiella pneumoniae* NCTC 13443 disinfectant-adapted samples. a) Fold enrichment of biological process GO terms. b) Fold enrichment of cellular component GO terms. Blue indicates a higher expression of proteins associated with the term, and red indicates a lower expression of proteins associated with the term, as depicted in the key. BAC: benzalkonium chloride-adapted samples. DDAC: didecyldimethylammonium chloride-adapted samples. PHMB: polyhexamethylene biguanide-adapted samples. CC: chlorocresol-adapted samples. BR: bronopol-adapted samples. The “glycolytic process” and “cytoplasm” GO terms are repeated due to individual samples showing both higher and lower expression of proteins associated with these terms.


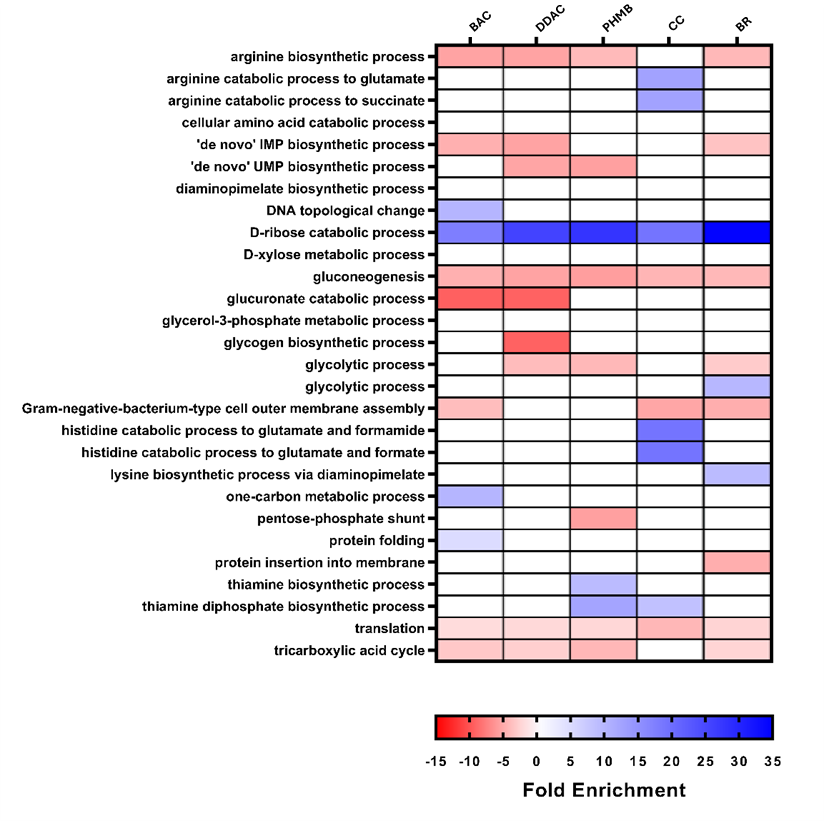

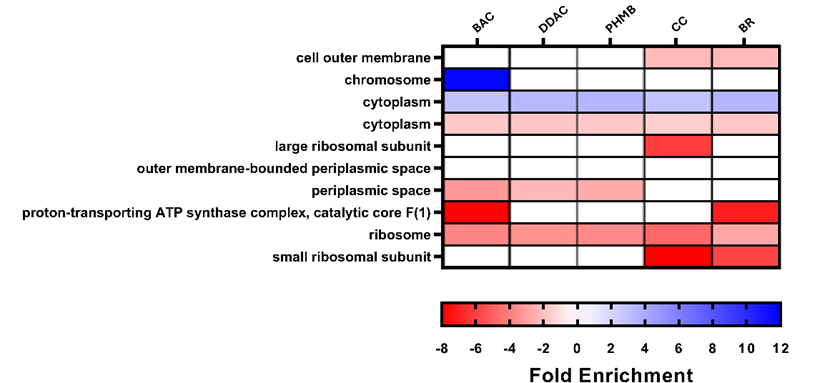


a)

b)
